# An invaluable transgenic blueberry for studying chilling-induced flowering in woody plants

**DOI:** 10.1186/s12870-018-1494-z

**Published:** 2018-11-01

**Authors:** Guo-qing Song, Aaron Walworth

**Affiliations:** Plant Biotechnology Resource and Outreach Center, Department of Horticulture, Michigan State University, East Lansing, MI 48824 USA

**Keywords:** Chilling requirement, Floral transition, Flowering time, *Vaccinium corymbosum* L., Dormancy release

## Abstract

**Background:**

Many deciduous woody crops require a minimum level of chilling to break dormancy and allow the seasonal growth of vegetative and floral buds. In this study, we report the discovery of an invaluable transgenic event of the blueberry cultivar ‘Legacy’ (hereafter, Mu-Legacy) for studying chilling-induced flowering in woody plants. Mu-legacy and its progeny provide a unique material to study the unknown mechanism of chilling-mediated flowering in woody plants.

**Results:**

Unlike nontransgenic ‘Legacy’ and plants of 48 other transgenic events, Mu-Legacy plants were able to flower under nonchilling conditions and had early flower bud formation, reduced plant size, and reduced chilling requirement for normal flowering. These characteristics were heritable and also observed in self-pollinated, transgenic T_1_ progenies of Mu-Legacy. A 47-Kbp genomic sequence surrounding the transgene insertion position was identified. RNA-sequencing data showed increased expression of a *RESPONSE REGULATOR 2*-like gene (*VcRR2*), located adjacent to the insertion position in Mu-Legacy and likely driven by the CaMV 35S promoter of the transgene. The Mu-Legacy showed 209 differentially expressed genes (DEGs) in nonchilled flower buds (compared to nontransgenic ‘Legacy’), of which only four DEGs were in the flowering pathway. This suggests altered expression of these few genes, *VcRR2* and four flowering DEGs, is sufficient to significantly change flowering behavior in Mu-Legacy.

**Conclusions:**

The significance of *VcRR2* in Mu-Legacy suggests that the *VcRR2*-involved cytokinin pathway likely contributes to the major differences in chilling-mediated flowering between woody and herbaceous plants. More importantly, Mu-Legacy shows increased yield potential, a decreased chilling requirement, and better winter hardiness than many low-chilling cultivars growing in southern warm winter conditions.

**Electronic supplementary material:**

The online version of this article (10.1186/s12870-018-1494-z) contains supplementary material, which is available to authorized users.

## Background

Deciduous fruit-bearing crops that grow in temperate climates come mainly from nine families, including *Rosaceae* (apples, pears, quinces, almonds, apricots, plums, cherries, peaches, raspberries, blackberries, loquats, and strawberries) [1], *Fagaceae* (chestnuts), *Betulaceae* (filberts), *Juglandaceae* (pecans and walnuts), *Ebenaceae* (persimmons), *Moraceae* (figs and mulberries), *Vitaceae* (grapes), *Ericaceae* (blueberries and cranberries), and *Grossulariaceae* (currants and gooseberries). These crops often require a certain period of cold exposure (chilling) to stimulate dormancy release and induce floral buds to blossom in their life cycles. Healthy flowering under the changing climate is of critical importance for sustainable production of temperate woody crops. Generally, the chilling requirement and plant hardiness, both of which vary among plant species, are the main consideration in regard to low temperatures when temperate woody crops are adopted.

*Vaccinium* is a genus of terrestrial shrubs in the family *Ericaceae* (Syn. Heath) containing approximately 450 species [2]. Highbush blueberries (*Vaccinium corymbosum* L.), including northern and southern ecotypes, are the major cultivated blueberries. Most of the commercial highbush cultivars require chilling to ensure normal flowering. The northern highbush cultivars require more than 800 chill units (CU) to break dormancy and flower in the spring and have generally better winter hardiness. In contrast, the southern highbush blueberry often needs 150 to 800 CU, but has greater high-temperature tolerance in the summer. Blueberry floral bud initiation often starts before endo-dormancy. Enough chilling accumulation during endo-dormancy is critical for floral bud formation and bud-break in the Spring for decidous woody fruit and nut crops. Insufficient chilling hours prevent bud-break and often lead to reduced fruit production. Manipulation of chilling requirement are considered to be long-term solution secure deciduous fruit production under the changing climate [3].

To date, extensive studies have elucidated the molecular basis of vernalization and flowering in *Arabidopsis* [4–12], the cereals [8, 9, 13] and beets [14]. In contrast, far less progress has been made in unveiling the molecular pathways of chilling-induced dormancy release in woody perennials, due mainly to the high complexity of their genomes. It has been suggested that overlap may exist between components of the herbaceous vernalization pathways and the dormancy release pathways of woody plants [15]. However, neither the *FRIGIDA* (*FRI*) [16] or *FLOWERING LOCUS C* (*FLC*)-determined [4, 5, 17] vernalization models in *Arabidopsis* nor the *VERNALIZATION* gene (*VRNs*)-mediated vernalization models in cereals and their relatives have proven sufficient to form a model of cold-dependant flowering in woody plants [8, 9, 13]. In general, the chilling requirement for dormancy-break is species- and genotype-dependent [14, 18].

Functional analysis of a blueberry *FLOWERING LOCUS T* (*FT*) gene (*VcFT*) has been conducted to study flowering mechanisms in highbush blueberry (*Vaccinium corymbosum* L.) [19–21]. Overexpression of *VcFT* (approx. 2900-fold increase in leaf tissues) caused continuous and precocious flowering in in vitro shoots and in one-year-old ‘Aurora’ plants [19]. However, two- and three-year-old *VcFT*-overexpressing plants did not flower normally, and the majority of the flower buds did not open under greenhouse conditions without chilling. It is interesting that overexpression of *VcFT* was not sufficient to completely release all blueberry floral buds from dormancy, suggesting that the molecular pathways for floral transition and breaking of seasonal dormancy only partially overlap in this species [20, 21].

Blueberry floral initiation occours before bud dormancy release. Transcriptome analysis using RNA sequencing data from nonchilled, chilled, and late pink buds of southern highbush blueberry ‘Legacy’ has revealed genes associated with chilling accumulation and bud break [3]. It is interesting that *VcFT* expression did not show a differential expression in chilled flower buds (compared to nonchilled flower bud) but were up-regulated in late-pink buds (compared to chilled flower bud. *DORMANCY ASSOCIATED MADS-box* (*DAM*) genes which are a cluster of six MADS-box transcription factors. The loss of all or part of the *DAMs* resulted in the non-vernalizaed peach *evergrowing* mutant [22, 23]. The *DAM* genes are considered alternatives to *FLC* in regulating vernalization-mediated chilling requirement and flowering [22, 24]. However, the *DAM* genes show high similarities to *A. thaliana AGAMOUS-LIKE 24* (*AGL24*) and *SHORT VEGETATIVE PHASE* (*SVP*) genes [24, 25]. Additionally, functional analysis of *DAMs* to reveal their roles in chilling-mediated flowering through reverse genetics has not been reported in peach. *VcSVP* showed differential expression in chilled and late-pink buds. In chilled blueberry flower buds, both *VcFLC* and *VcSVP* homologues decreased but increased in late-pink buds [3]. The functional orthologues of *FLC* and *AGL24* were not detected in blueberry, suggesting that the vernalization/chilling-mediated flowering pathway of blueberry is different from *A. thaliana.*

Expression of blueberry *DWARF AND DELAYED FLOWERING* gene (*VcDDF1*) is not cold-inducible; however, overexpression of the *VcDDF1* increases freezing tolerance in transgenic leaves and flower buds [26–28]. One transgenic event of blueberry ‘Legacy’ (herein Mu-Legacy) transformed with *VcDDF1* was identified from 49 transgenic events because it can flower under nonchilling conditions whereas all the nontransgenic and transgenic plants of 48 other events could not. This Mu-Legacy is a desirable material for revealing the mechanism of chilling-induced flowering in woody plants.

## Results

### Phenotypes of mu-legacy

**Fig. 1:**
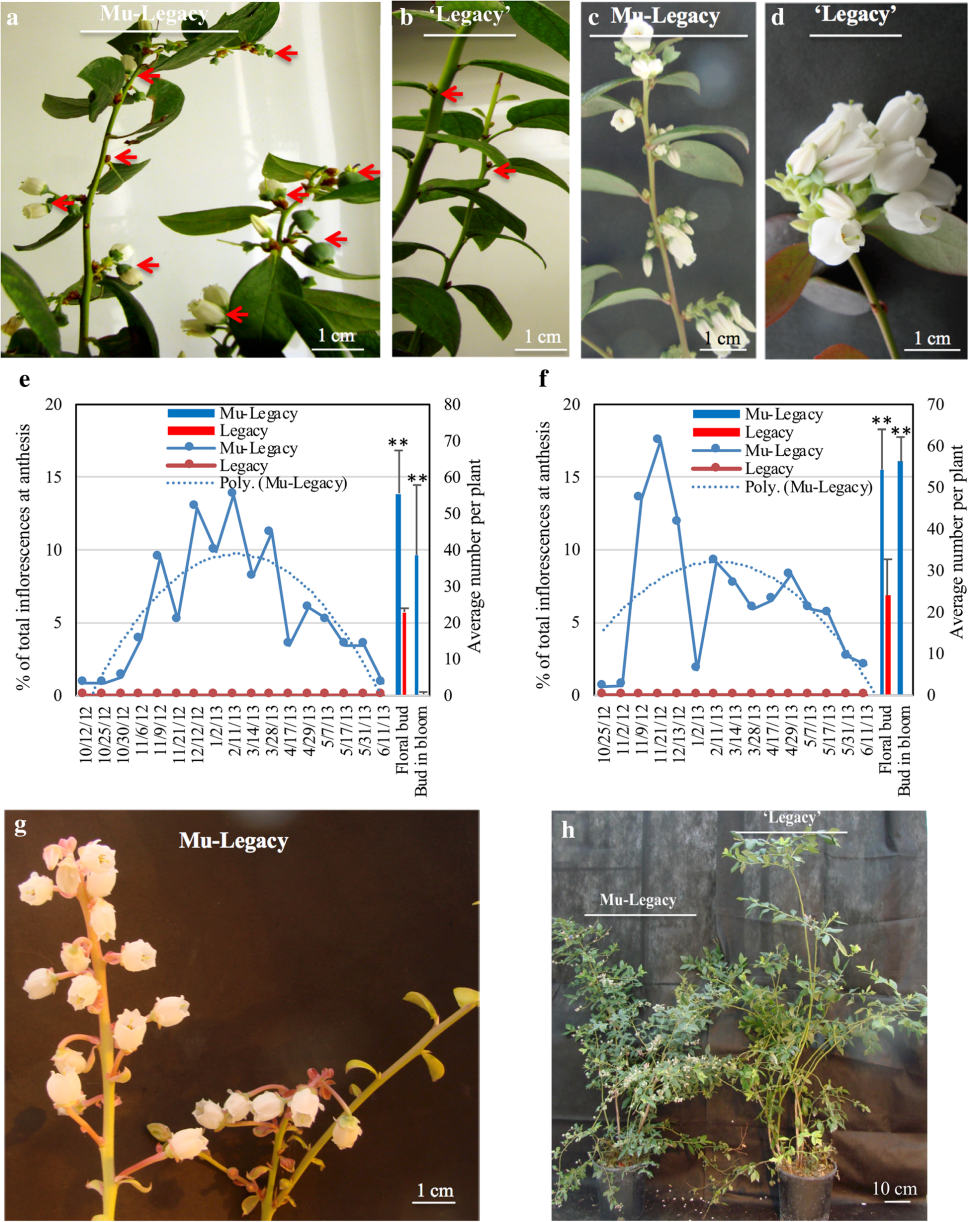
Flowering of Mu-Legacy and nontransgenic ‘Legacy’ plants. **a**, **b**, Flowering of two-year old, nonchilled Mu-Legacy (**a**) and ‘Legacy’ (**b**) plants under a short-day photoperiod (nine hours). Red arrows indicate flowers, fruits, or flower buds. **c**, **d**, Flowering of fully chilled two-year old Mu-Legacy (**c**) and ‘Legacy’ (**d**) plants under a long-day photoperiod (14 h). **e**, **f**, Pattern of flowering when three-year old Mu-Legacy and ‘Legacy’ plants (*n* = 6) were grown in greenhouses under nonchilling conditions with a short-day photoperiod (nine hours) (**e**) and a long-day photoperiod (16 h) (**f**). The primary y-axis is for the line chart and the secondary y-axis is for the column chart. The bars are showing the mean number of flower buds per plant and the number of flower buds that bloomed. Each data point is an average of six plants plus standard deviation bars. **g**, In addition to the flowering pattern observed under a short-day photoperiod (nine hours) (**a)**, flowering was shown on the new shoots of Mu-Legacy under a long-day photoperiod (16 h). **h**, A shorter Mu-legacy plant is showing more fruit production than a nontransgenic ‘Legacy’ plant. The five-year old plants were grown in a secured courtyard under natural environmental conditions. Significance (compared to nontransgenic ‘Legacy’) determined using a Student’s *t*-test is denoted. One asterisk (*) indicates *p* < 0.05 and two asterisks (**) indicate *p* < 0.01

Sixteen nontransgenic and 202 transgenic ‘Legacy’ plants were grown in a heated greenhouse in Michigan in 2009. These plants were developed from in vitro shoot cultures and were grown in the greenhouse for approximately 14 months after they were rooted. The transgenic plants, three to five plants per transgenic event, were from 49 transgenic events, each containing an overexpression vector for *VcDDF1* [27, 28]. All three plants of one *VcDDF* transgenic event, which was designated Mu-Legacy, started flowering in the heated greenhouse in mid-October (Fig. 1a, b), while all 16 nontransgenic ‘Legacy’ and 199 transgenic plants of the other 48 *VcDDF1* transgenic Legacy events could not flower without chilling. The Mu-Legacy had an obviously shortened juvenility phase, and could flower without the typical dormancy period and subsequent release following chilling. These results suggest that the overexpressed *VcDDF1* is probably not the only factor causing the changes in Mu-Legacy plants. As of this report, 200 plants for each of the Mu-Legacy and nontransgenic ‘Legacy’, and 100 plants of one representative transgenic event (hereafter, *VcDDF1*-Legacy) of the other 48 *VcDDF1* transgenic events were obtained through micropropagation for continuous investigation over the previous five years.

Flowering behaviors of chilled Mu-Legacy plants are similar to the normal flowering of chilled nontransgenic ‘Legacy’ (Fig. 1c, d) and transgenic plants of the 48 *VcDDF1* transgenic events (data not shown). Nonchilled Mu-Legacy plants had a flowering period of six to seven months, regardless of photoperiod (e.g., October 21, 2009 to May 05, 2010) (Fig. 1e, f), and each inflorescence had one to four flowers at a time (Fig. 1a); in contrast, chilled Mu-Legacy plants with approximately 1000 CU had a flowering period of approximately 10 days and each flower bud had six to 10 flowers (Fig. 1d). The difference in flowering between long-day and short-day grown nonchilled Mu-Legacy plants is that the long-day grown plants showed flowered on newly formed soft-wood shoots, which was not observed in plants grown under short day (Fig. 1g). These results suggest that compared to nontransgenic ‘Legacy’ the Mu-Legacy plants have a mutation that contributes to altered chilling requirment (Fig. 1). However, the Mu-Legacy still requires some accumulation of chilling for normal flowering.

**Fig. 2:**
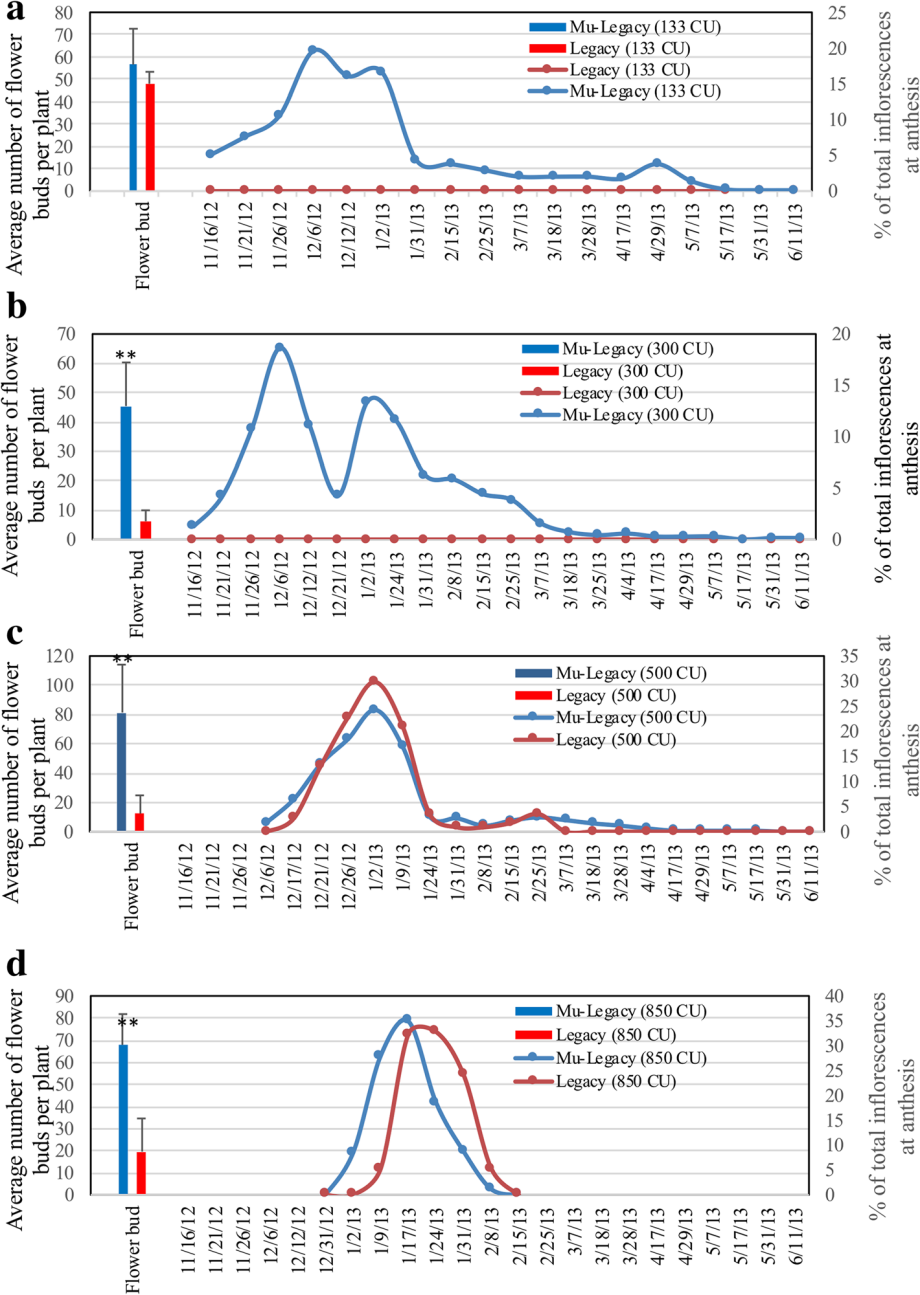
Response of three-year old Mu-Legacy and nontransgenic ‘Legacy’ plants to different chilling hours in the greenhouse at 22–24 °C under natural light conditions. These plants (*n* = 5) were grown under normal chilling conditions (> 1000 chilling hours) before they reached three-years old. The primary y-axis is for the column chart and the secondary y-axis is for the line chart. Each data point is a mean of five plants. Significant changes determined using a Student’s *t*-test are denoted. Asterisk (**) indicates *p* < 0.01

When looking at one year-old plants with over 1000-CU of cold exposure, none of the nontransgenic ‘Legacy’ plants flowered, wheras 84.9% of Mu-Legacy plants flowered. This indicates early floral bud formation in the Mu-Legacy plants with a reduced juvenility phase. Chilling accumulation influences the bloom period of both Mu-Legacy and nontransgenic ‘Legacy’ plants. For Mu-Legacy, lower levels of chilling accumulation (133 or 300 CU) were associated with longer bloom periods when compared with greater chilling accumulation (500 or 850 CU) (Fig. 2). Flowering of nontransgenic ‘Legacy’ plants was not observed until chilling accumulation reached 500 CU (Fig. 2c). With 850-CU chilling, Mu-Legacy and nontransgenic ‘Legacy’ plants showed a similar, very condensed bloom period, but Mu-Legacy flowered two days earlier than nontransgenic ‘Legacy’ plants (Fig. 2d).

**Fig. 3:**
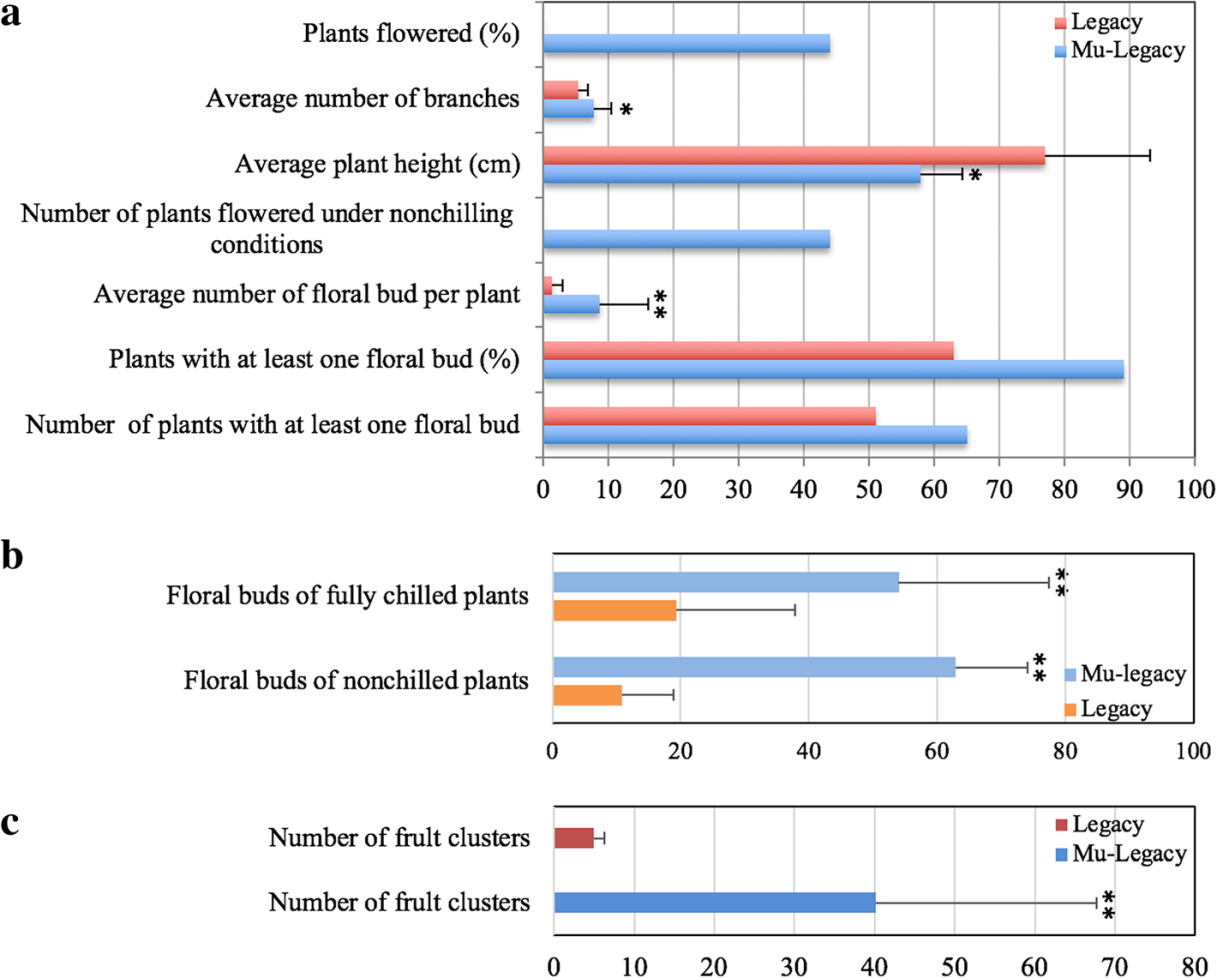
Architectures, flowering, and fruit production of Mu-Legacy and nontransgenic ‘Legacy’ plants. **a,** Architectures and flowering of two-year old ‘Mu-Legacy’ and nontransgenic ‘Legacy’ plants. Plants (100 plants for each of nontransgenic ‘Legacy’ and ‘Mu-Legacy’ were investigated). Red arrows show flowers or fruits. **b**, Inflorescence bud formation in chilled and nonchilled, three-year old Mu-Legacy and nontransgenic ‘Legacy’ plants (*n* = 12). **c**, Fruit clusters for six-year old Mu-Legacy and nontransgenic ‘Legacy’ plants (*n* = 5) grown under natural environmental conditions after full chilling in winter in 2017. Significance determined using a Student’s *t*-test is denoted. One asterisk (*) indicates *p* < 0.05 and two asterisks (**) indicate *p* < 0.01

Mu-Legacy had a smaller plant size, greater flower bud formation, and a reduced chilling requirement when compared to either nontransgenic ‘Legacy’ (Fig. 1e,f; Figs. 2, 3) or the transgenic *VcDDF1*-Legacy plants. These changes resulted in a high yield potential for Mu-Legacy plants grown under both nonchilling and fully-chilled conditions.

### Phenotypes of the T_1_ progeny of mu-legacy

**Fig. 4:**
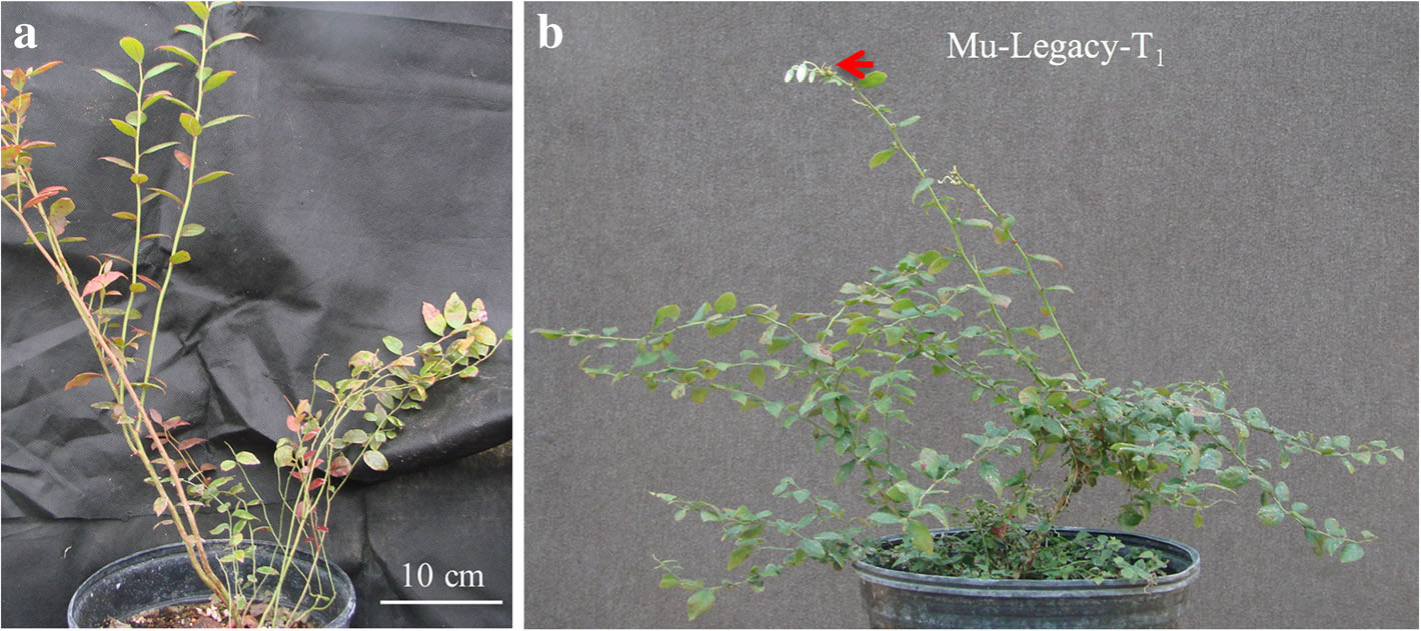
Two-year old T_1_ plant of Mu-Legacy grown under nonchilling conditions. **a**, A nontransgenic T_1_. **b**, Mu-Legacy-T_1_ (an extreme dwarf, transgenic T_1_ plant). The arrow shows flowers

The chilling-independent flowering trait in Mu-Legacy was heritable and segregated in the T_1_ generation. Of 36 self-pollinated T_1_ seedlings of Mu-Legacy grown in a heated greenhouse for two years, 22 plants were able to flower prior to their exposure to any chilling while 14 plants did not flower (Additional file 1: Fig. S1). All of these 22 T_1_ plants were PCR-positive for both the neomycin phosphotransferase II (*npt*II) and *VcDDF1* transgenes; in contrast, none of the 14 non-flowering plants were PCR-positive. The transgene segration rate is 3:1 (chi square test, *p* < 0.01). Other phenotype variations, such as plant size, architecture, and leaf shape and size, were also observed in the 36 T_1_ plants. One extremely dwarf transgenic seedling (herein Mu-Legacy-T_1_) was identified (Fig. 4). These results suggest that the tranfer DNA (T-DNA) insertion is responsible for the phenotypic changes in the Mu-Legacy.

### Genetic control of the mutation in mu-legacy

An electrolyte leakage assay suggested that overexpressing *VcDDF1* resulted in a significant increases in freeze tolerance in leaf tissues of several transgenic events, i.e., the representative transgenic event *VcDDF1*-Legacy (previous code: II7) but not in Mu-Legacy (previous code: II3) [28]. In compared to nontransgneic ‘Legacy’, *VcDDF1* overexpression (*VcDDF1*_OX) in Mu-Legacy resulted in increases in *VcDDF1* expression (c32575_g1_i1) at 108-fold in nonchilled flower buds (Additional file 2: Table S1) and 40-fold in leaves (Additional file 3: Table S2), respectively. In compared to *VcDDF1*_Legacy, the *VcDDF1*_OX in Mu-Legacy did show differential expression in nonchilled flower buds (Additional file 4: Table S3) but showed a significant decrease in leaves (Additional file 5: Table S4). Thus, little evidence supports that the overexpressed *VcDDF1* is the only explanation for the mutations in Mu-Legacy.

**Fig. 5:**
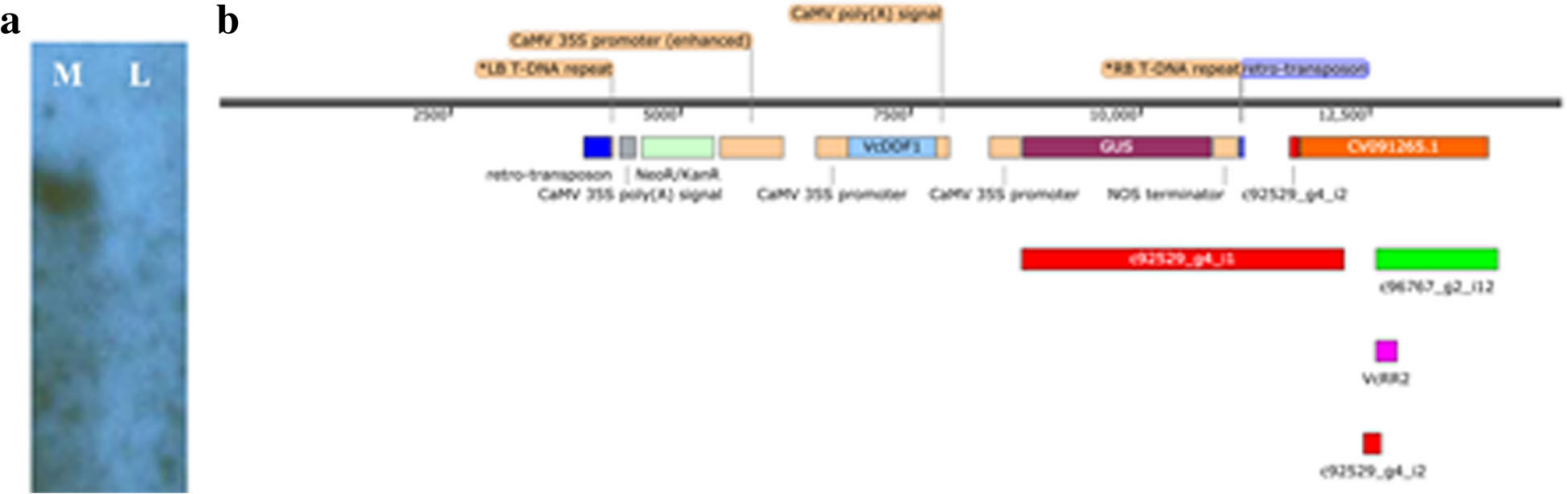
Transgene insertion position in Mu-Legacy and its effect on expression of adjacent gene(s). **a**, Southern blot analysis of Mu-Legacy (M) and Legacy (L). About 30 micrograms DNA was digested using *Hin*dIII. The *gus*A coding region was used as the probe. **b**, The T-DNA was inserted into a blueberry retro-transposon region, which locates at the upstream of *VcRR2*. c92529_g4_i1, c92529_g4_i2, c96767_g2_i12 are the transcripts near the right repeat of the T-DNA insertion region. *LB T-DNA repeat: there was a deletion of 18 from 5′-end. *RB T-DNA repeat: this repeat (25 bp) and 37 bp up its 5′-end, including 16 bp belonging to the NOS terminator, were deleted. The insertion increased overexpression of the *VcRR2* in gene contigs c92529_g4 and c96767_g2 in both leaf and flower bud tissues of Mu-Legacy and also in leaf tissues of Mu-Legacy-T_1_ (Table 1)

Southern blot analysis indicated that Mu-Legacy contains one copy of the T-DNA (Fig. 5a). The T-DNA insertion position was initially found in a 1279 base pairs (bp) fragment. The left border of the inserted T-DNA has an 18-bp deletion, and the right border has a 68-bp deletion. The insertion broke a 354-bp reading frame, which shows the highest similarity at protein level to a retrotransposon protein of rice (*Oryza sativa*). Sequence analysis of RT-PCR products showed the presence of the 354-bp fragment in the leaf samples of both Mu-Legacy and nontransgenic ‘Legacy’, suggesting that the 354-bp sequence has at least two identical alleles in nontransgenic ‘Legacy’. Using the 1279 bp sequence of the T-DNA insertion position of Mu-Legacy to search blueberry ESTs, we found a 781 bp EST contig CV091265.1 that has 199 bp overlap with the 1279 bp sequence. The 199 bp region (herein *VcRR2*) of this EST shows a high similarity to B-type *RESPONSE REGULATOR 2* (*ARR2*). Based on this EST sequence, the sequence at the insertion position could be extended to 3053 bp (Additional file 6 Supplemental information 1); however, the sequence information alone is insufficient to explain why the insertion is responsible for the changes in Mu-Legacy.

### Genomes of mu-legacy and mu-legacy-T_1_

Full genome sequencing with an approximate 39-fold coverage generated high quality reads of 313 million reads (MR) of 94.0 G base pairs (Gbp) for Mu-Legacy and 313 MR of 93.3 Gbp for Mu-Legacy-T_1_, respectively. Each genome was assembled twice by using SOAPdenovo2 and ABySS/1.9.0, respectively. Using the 3053 bp sequence of the T-DNA insertion position of Mu-Legacy to search the assembled Mu-Legacy and Mu-Legacy-T_1_ genome databases led to an assembly of a maximum of 47 kilobase pair (Kbp) transgene insertion region (Fig. 5b).

### Four DEGs of the flowering pathway in mu-legacy

**Fig. 6:**
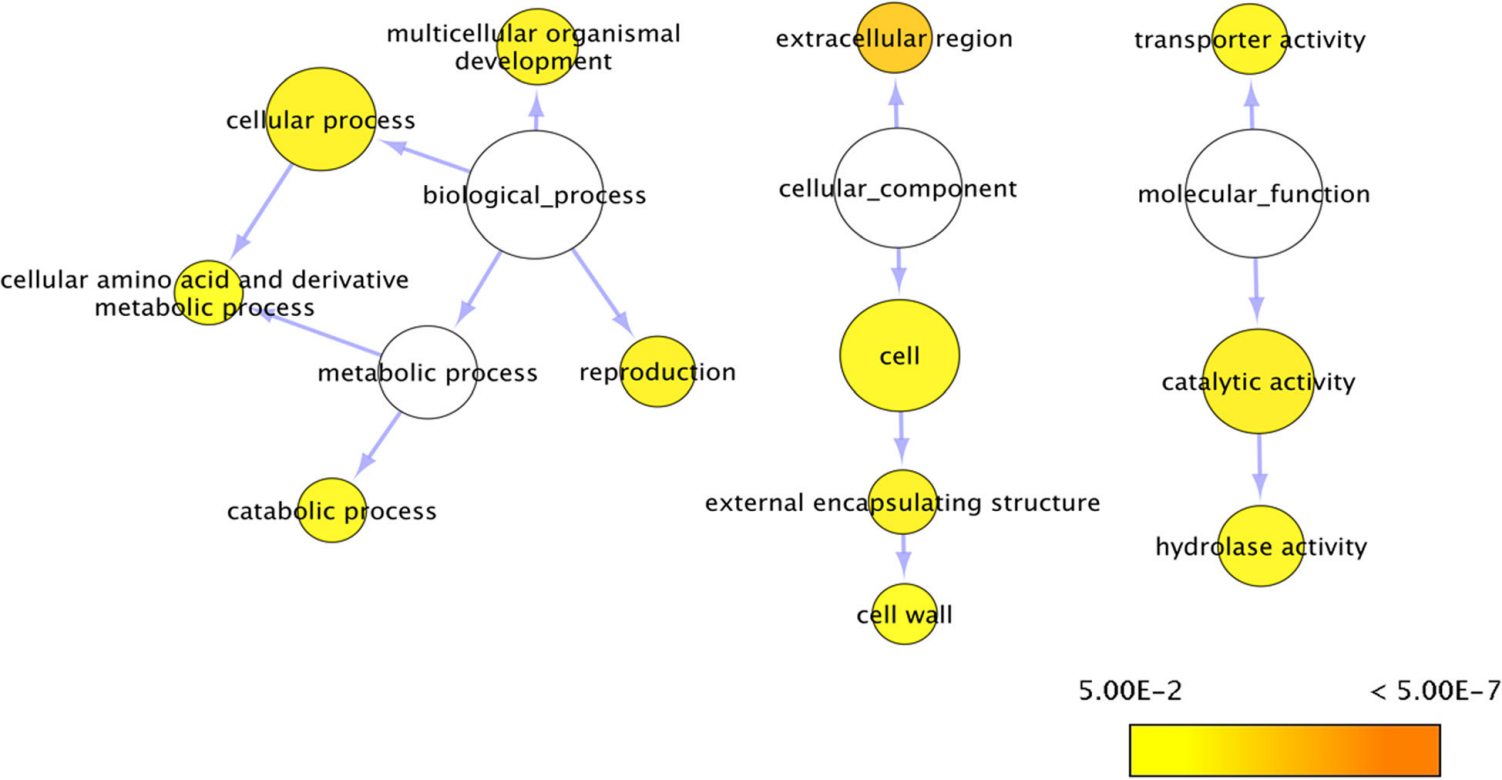
Gene networks of differentially expressed genes in nonchilled flower buds of Mu-Legacy plants. The gene ontology file of GOSlim_Plants in BiNGO was used to identify overrepresented GO terms (*P* < 0.05). Bubble size and color indicate the frequency of the GO term and the *P*-value, respectively

**Fig. 7:**
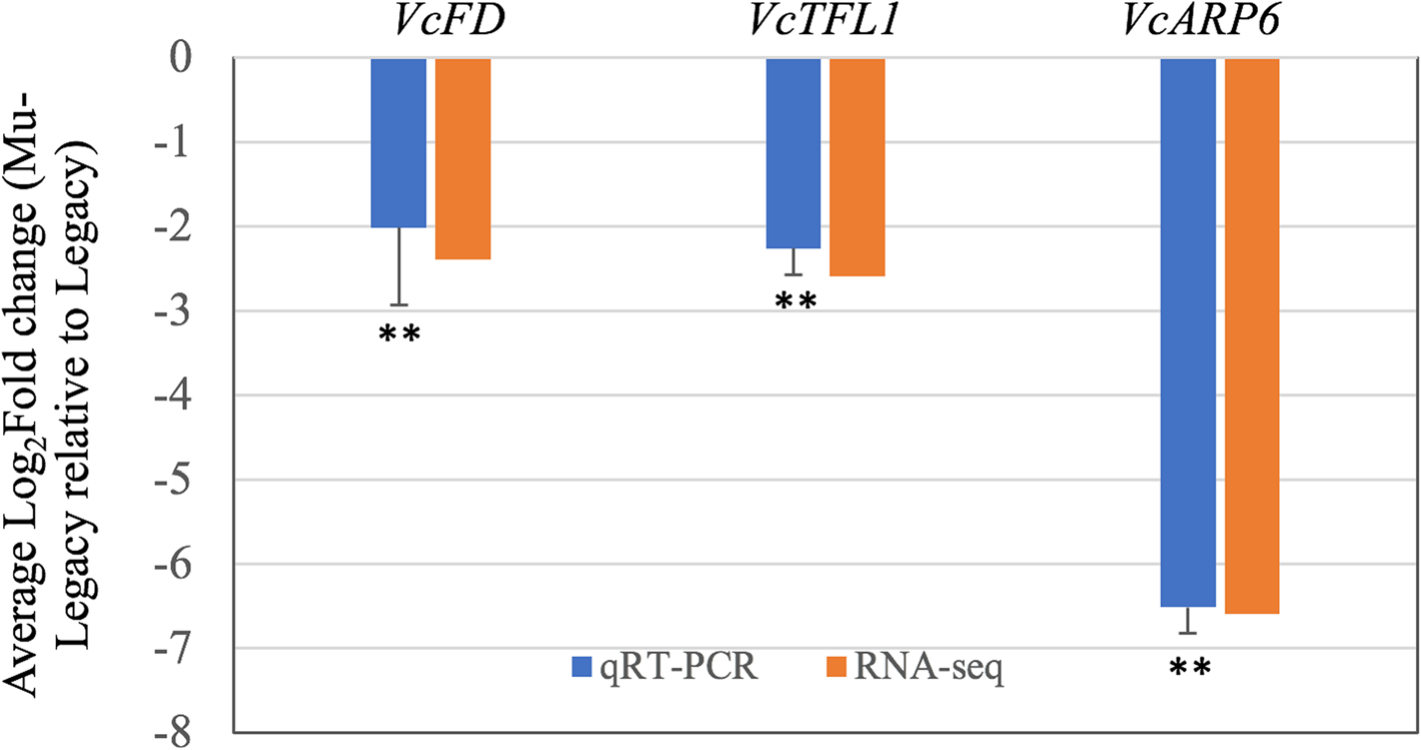
Quantative RT-PCR analysis of three differentially expressed flowering pathway genes (identified in RNA-seq with FDRs < 0.05; expression of *DOF5.3* was not tested) in nonchilled flower buds. Eukaryotic translation initiation factor 3 subunit H is the internal control. Relative expression Log_2_(fold-change) was calculated by -∆∆Ct = −[(Ct_GOI_ – Ct_nom_)_nonchilled Mu-Legacy_ – (Ct_GOI_ – Ct_nom_)_Legacy_]. Average fold-changes ± standard deviation of three biological replicates. Significant average Log_2_fold-change was determined using the Student’s test. Asterisks (**) indicate *p* < 0.01. RNA-seq data show Log_2_Fold change (Mu-Legacy/Legacy)

RNA sequencing data were generated for comparative analysis of nontransgenic‘Legacy’ and Mu-Legacy. The overrepresented gene ontology (GO) term “reproduction” in gene networks of the annotated DE isoforms supports the changes in flowering seen in Mu-Legacy plants (Fig. 6). The DE gene networks of Mu-Legacy have much less GO terms than those of *VcDDF1*-Legacy [27], indicating the potential effects of both insertion positions and *VcDDF1* expression levels. When flowering pathway genes of *A. thaliana* were used to identify DE genes (DEGs), only four DEGs, including orthologues of *PROTEIN FD (FD), TERMINAL FLOWER 1 (TFL1)*, *ACTIN-RELATED PROTEIN6* (*ARP6*), *DOF ZINC FINGER PROTEIN5.3* (*DOF5.3*), were detected and they were all repressed (Fig. 7, Additional file 2: Table S1). These results suggest that the four DEGs of the flowering pathway in Mu-Legacy play significant roles in chilling-mediated flowering.

### DEGs of major phytohormone-related genes and COR genes in mu-legacy

Due to the potential effect of phytohormones in chilling-mediated blueberry flowering [3], DE orthologues of five major phytohormones of *A. thaliana* [i.e., abscisic acid (ABA), gibberellic acid (GA), auxin, cytokinin, and ethylene] were examined in Mu-Legacy tissues (Additional file 2: Tables S1, Additional file 3: Tables S2). GA is involved in the flowering pathway through its interaction with the *SUPPRESSOR OF OVEREXPRESSION OF CONSTANS 1* (*SOC1*) and *TFL1* [29, 30]. In nonchilled flower buds, orthologues of *ENT*-*KAURENOIC ACID HYDROXYLASE* (*KAO2*) and *GA REQUIRING 3* (*GA3*) in the GA biosynthesis pathway were both upregulated (|Log_2_FC = Log_2_fold changes | < 2) without being associated with a differential expression of blueberry *SOC1* (*VcSOC1*). DE orthologues of the *CYP83B1 PROTEIN* (*SUR2*) in the auxin pathway were also upregulated. DE orthologues of two *A. thaliana* genes in the cytokinin pathway, one in the ethylene pathway, and one in the auxin pathway showed low fold changes (|Log_2_FC| < 2). Compared to the number in nonchilled flower buds (eight in total), fewer DE orthologues of these phytohormone genes were present in leaves (two) (Additional file 3: Table S2). These DE phytohormone genes in different tissues could play a role in the early flowering and reduced size of Mu-Legacy plants.

DE orthologues of *A. thaliana* cold-regulated genes (CORs) were present in Mu-Legacy plants (Additional file 2: Tables S1, Additional file 3: Tables S2). No significant increase in freezing tolerance was observed in leaf and bud tissues in the electrolyte leakage assay (previous code: II3) [28], suggesting that these DE CORs were insufficient to drive a significant increase in freezing tolerance.

### DEGs caused by insertion position

**Fig. 8:**
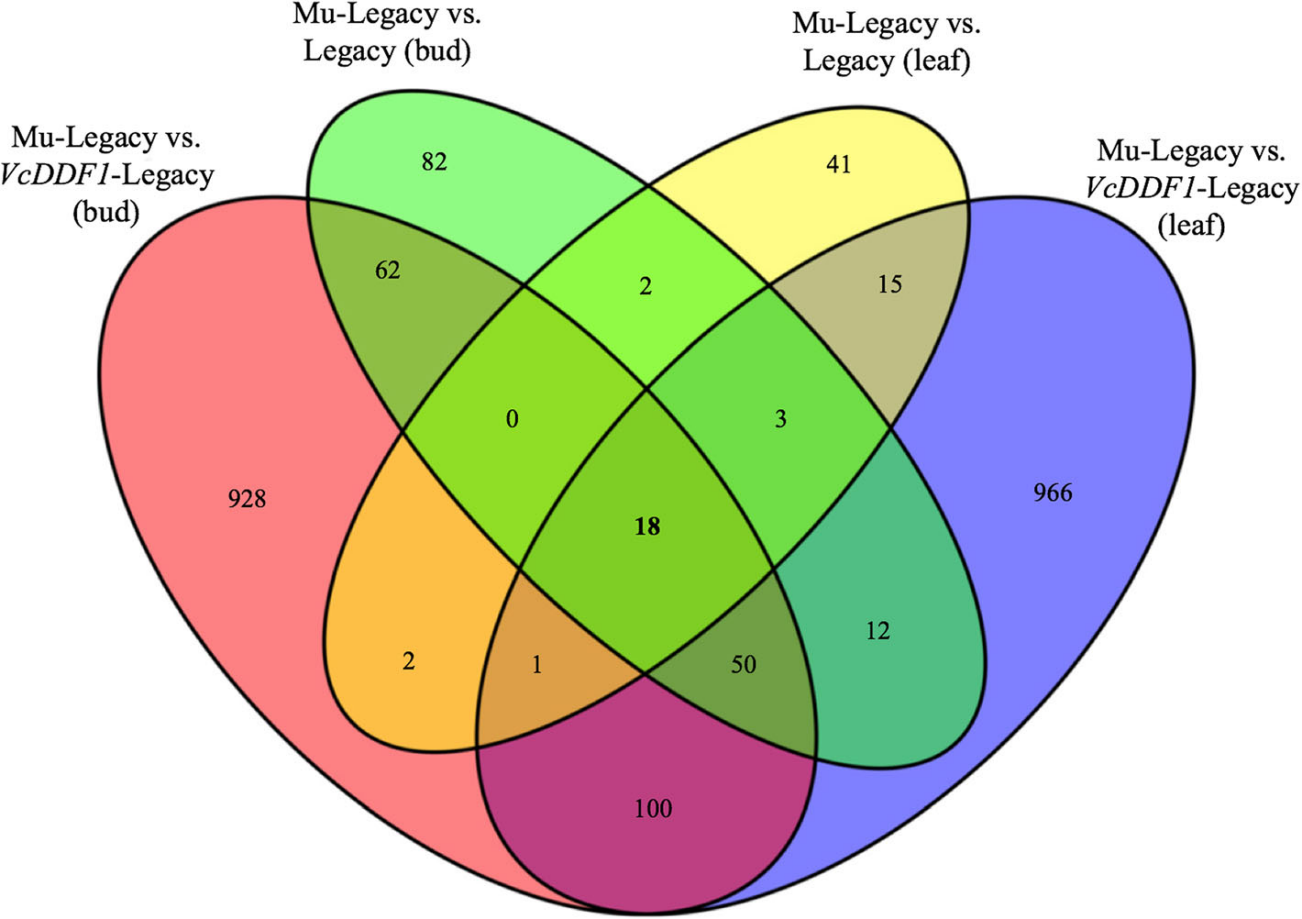
Differentially expressed genes in four transcriptome comparisons. The numbers show DE unique genes (based on the annotation using Trinotate)

Unlike Mu-Legacy plants, *VcDDF1*-Legacy plants, flowered similar to nontransgenic ‘Legacy’ [27], suggesting that the overexpressed *VcDDF1* might not be the major factor for the altered flowering in the Mu-Legacy. To further explore the potential candidate genes that could be resposible for the mutation in Mu-Legacy, transcriptomic analyses of Mu-Legacy (compared to *VcDDF1*-Legacy) were also conducted in leaves and nonchilled flower buds. The comparisons resulted in 1108 and 1119 DE unique genes in leaves and nonchilled flower buds, respectively (Additional file 4: Table S3, Additional file 5: Table S4). Further analysis of the four groups of DEGs identified in the comparions of Mu-Legacy vs. nontransgenic ‘Legacy’ (77 for leaves and 209 for flower buds) and Mu-Legacy vs. *VcDDF1*-Legacy (1108 for leaves and 1119 for flower buds) resulted in 18 shared DEGs and 31 unannotated transcripts by Trinotate, which were the major DEGs and DE transcripts caused by the transgene insertion position in Mu-Legacy (Fig. 8; Table 2, Additional file 2: Table S1, Additional file 3: Table S2, Additional file 4: Table S3, Additional file 5: Table S4). The 18 DEGs and 31 DE transcripts were further annotated by searching the Arabidopsis protein database (Table 2). Of the 18 DEGs, the upregulated c96767_g2_i12 is a *VcRR2* gene adjacent to the insertion position in Mu-Legacy (Fig. 5, Tables 1 and 2). The repressed c49456_g1_i1 and c49456_g2_i2 (annotated to ACTS_RAT or actin-11) showed a high similarity to the *Arabidopsis ARP6* gene, which regulates *FLC* independent of vernalization *in Arabidopsis* [31] Thus, the upregulated *VcRR2* and the repressed *ARP6* could be the key DEGs causing the changes (compared to both nontransgenic ‘Legacy’ and *VcDDF1*-Legacy) in chilling-mediated flowering in the Mu-Legacy. It appears from this data that a small numbers of DEGs were responsible for the phenotypic changes in the Mu-Legacy (Fig. 1-3), suggesting that the Mu-Legacy is an invaluable material to study the blueberry flowering mechanism.

**Table 1:** Differentially expressed transcripts (*VcRR2*) adjacent to the transgene insertion position in Mu-Legacy and Mu-Legacy-T_1_ plants

	Isoform ID	logFC	logCPM	*P*-Value	FDR
Mu-Legacy (leaf)					
	c92529_g4_i1	2.84	5.64	8E-18	1E-13
	c92529_g4_i2	6.17	5.04	3E-36	7E-32
	c96767_g2_i12	4.40	2.25	1E-17	2E-13
Mu-Legacy (bud)					
	c92529_g4_i1	4.10	5.52	2E-61	9E-57
	c92529_g4_i2	7.26	5.32	2E-98	1E-93
	c96767_g2_i12	4.22	2.64	7E-35	1E-30
Mu-Legacy-T_1_ (leaf)					
	c92529_g4_i1	2.24	5.75	2E-82	4E-79
	c92529_g4_i2	5.59	5.30	4E-227	8E-223
	c96767_g2_i12	4.35	3.13	9E-71	1E-67

*FDR* false discovery rate. *LogFC* Log_2_(fold change) = Log_2_[Mu-Legacy (or Mu-Legacy-T_1_)/nontransgenic Legacy]. LogCPM: Log_2_(counts per million)

**Table 2:** Differentially expressed genes and transcripts shared in four transcriptome-comparisons

Transcript_ID	Log_2_Fold change (Mu-Legacy/Legacy bud)	Log_2_Fold change (Mu-Legacy/Legacy leaf)	Log_2_Fold change (Mu-Legacy/VcDDF1-Legacy bud)	Log_2_Fold change (Mu-Legacy/VcDDF1-Legacy leaf)	Annotated by Trinotate	Top hit to Arabidopsis proteins	E Value in the blastp to Arabidopsis	Annotation in Arabidopsis
c74717_g1_i1	−7.81	−6.45	− 7.88	−5.91	AT2A1_BOVIN	AT1G10130.1	0E + 00	endoplasmic reticulum-type calcium-transporting ATPase 3
c46791_g1_i1	−7.20	− 7.80	− 7.51	−7.70	G3P_FELCA	AT1G16300.1	3E-173	glyceraldehyde-3-phosphate dehydrogenase of plastid 2
c85583_g3_i1	7.47	9.70	7.01	9.52	.	AT1G22110.3	3E-17	structural constituent of ribosome
c96767_g2_i1	4.13	3.73	4.12	3.92	.	AT1G72700.3	1E-07	ATPase E1-E2 type family protein
c96767_g2_i10	4.32	4.22	4.33	3.61	.	AT1G72700.3	9E-08	ATPase E1-E2 type family protein
c96767_g2_i12 (c92529_g4_i2)	4.22	4.32	4.85	4.00	. (ARR2_ARTH)	AT1G72700.3	9E-08	ATPase E1-E2 type family protein
c96767_g2_i2	2.96	2.65	3.58	2.92	.	AT1G72700.3	1E-07	AT1G72700.3 | ATPase E1-E2 type family protein
c96767_g2_i6	2.52	2.29	3.58	2.30	.	AT1G72700.3	1E-07	ATPase E1-E2 type family protein
c96767_g2_i9	2.38	2.89	3.12	2.75	.	AT1G72700.3	1E-07	ATPase E1-E2 type family protein
c96767_g2_i7	3.27	3.12	3.97	3.39	.	AT1G72700.3	1E-07	ATPase E1-E2 type family protein
c85583_g2_i2	6.48	8.45	5.20	7.14	.	AT1G77932.1	2E-12	FANTASTIC four protein%2C putative (DUF3049)
c87534_g1_i2	−7.59	−8.52	− 8.25	−7.45	TITIN_HUMAN	AT2G17033.4	6E-01	pentatricopeptide (PPR) repeat-containing protein
c96767_g2_i4	3.88	5.36	3.27	3.35	.	AT2G25100.1	4E-03	Polynucleotidyl transferase%2C ribonuclease H-like superfamily protein
c62793_g1_i1	−6.68	−8.63	−7.12	−7.78	ENOB_BOVIN	AT2G36530.1	0E + 00	Enolase
c88421_g5_i1	9.75	9.02	8.46	10.06	.	AT2G46240.1	7E + 00	BCL-2-associated athanogene 6
c49456_g1_i1	−7.90	−9.12	−8.28	−7.79	.	AT3G12110.1	1E-87	actin-11
c49456_g2_i2	−6.89	−5.98	−7.33	− 6.17	ACTS_RAT	AT3G12110.1	8E-164	actin-11
c99927_g1_i1	−8.10	−7.29	−8.39	− 7.11	.	AT3G19960.3	8E-162	myosin 1
c99927_g1_i2	−6.99	−8.12	−7.55	−7.78	.	AT3G19960.3	8E-162	myosin 1
c69050_g1_i1	−6.83	−5.82	−7.86	−7.50	.	AT3G20993.1	1E + 00	low-molecular-weight cysteine-rich 56
c76484_g1_i2	3.60	4.27	4.41	4.39	.	AT3G42860.1	4E-04	zinc knuckle (CCHC-type) family protein
c74413_g1_i1	−7.16	−6.35	−7.40	−5.88	PYGM_RABIT	AT3G46970.1	0E + 00	alpha-glucan phosphorylase 2
c92529_g4_i1	4.10	2.70	1.42	2.50	BGLR_ECOLI	AT3G54440.1	1E-25	glycoside hydrolase family 2 protein
c92529_g4_i4	10.37	9.18	1.30	11.64	.	AT3G54440.2	1E-08	glycoside hydrolase family 2 protein
c35591_g1_i2	−9.20	−7.84	−9.79	−7.67	TNNC2_RABIT	AT3G56800.1	3E-35	calmodulin 3
c64973_g1_i2	−7.65	−6.53	−8.20	−6.36	MYL1_RABIT	AT3G56800.1	3E-32	calmodulin 3
c64740_g1_i1	−7.36	−8.72	− 7.86	−7.67	LDHA_PIG	AT4G17260.1	1E-108	Lactate/malate dehydrogenase family protein
c44157_g1_i1	−7.82	−6.34	−8.28	−7.20	MYG_HORSE	AT4G21940.2	4E + 00	calcium-dependent protein kinase 15
c58218_g1_i2	−8.03	−7.01	− 8.31	−6.90	.	AT4G26530.3	5E-142	Aldolase superfamily protein
c58218_g1_i1	−8.75	−6.60	−9.02	− 6.66	ALDOA_HUMAN	AT4G26530.3	3E-141	Aldolase superfamily protein
c142707_g1_i1	6.22	6.88	3.50	5.59	.	AT4G31570.5	4E-01	nucleoporin
c55223_g1_i3	−7.57	−6.25	−7.98	−6.25	.	AT4G35800.2	7E + 00	RNA polymerase II large subunit
c117113_g1_i1	−7.38	−9.43	− 7.92	−8.37	MLRS_RABIT	AT5G21274.1	4E-15	calmodulin 6
c79880_g1_i1	−6.71	−6.30	−7.00	−6.08	TPM1_RAT	AT5G27950.1	3E + 00	P-loop containing nucleoside triphosphate hydrolases superfamily protein
c72151_g1_i1	−6.29	−10.53	−6.54	−9.36	KCRM_PIG	AT5G36670.1	2E + 00	RING/FYVE/PHD zinc finger superfamily protein
c64973_g1_i1	−5.22	−8.68	−5.95	−6.94	MYL1_BOVIN	AT5G37780.1	9E-30	calmodulin 1
c88158_g4_i2	−4.15	−3.57	−2.65	−2.48	.	AT5G43270.3	7E-01	squamosa promoter binding protein-like 2
c139587_g1_i1	5.25	7.29	5.20	6.07	.	AT5G62270.2	9E-10	ribosomal protein L20
c58774_g1_i1	−8.73	−5.95	−9.52	−7.49	.	ATCG01100.1	2E-30	NADH dehydrogenase family protein
c41067_g1_i1	−7.73	−6.75	−8.81	− 8.24	.	ATMG00160.1	1E-43	cytochrome oxidase 2
c70295_g1_i1	−8.44	−6.80	−9.61	−8.20	.	ATMG00220.1	1E-101	apocytochrome b
c82557_g1_i1	−6.45	− 6.25	−7.48	−7.82	NU6M_HORSE	ATMG00513.1	3E-71	NADH dehydrogenase 5A
c77169_g1_i1	−10.17	−7.95	−11.05	−9.09	.	ATMG00580.1	2E-28	NADH dehydrogenase subunit 4
c72780_g1_i1	−6.90	−5.76	−8.13	−7.44	.	ATMG00730.1	4E-69	cytochrome c oxidase subunit 3
c65695_g1_i1	−7.66	−6.33	−8.65	− 7.78	.	ATMG01360.1	0E + 00	cytochrome oxidase
c55223_g1_i1	−7.64	−6.50	−8.04	−6.01	.	No hits found	.	.
c70752_g2_i1	6.13	7.75	5.40	6.44	.	No hits found	.	.
c86721_g2_i2	−2.01	−2.54	−2.21	−2.34	.	No hits found	.	.
c70752_g1_i1	4.41	5.58	4.57	8.68	.	No hits found	.	.

### Blueberry B-type *RESPONSE REGULATOR* (*VcRR2*) is likely responsible for the mutation of mu-legacy

**Fig. 9:**
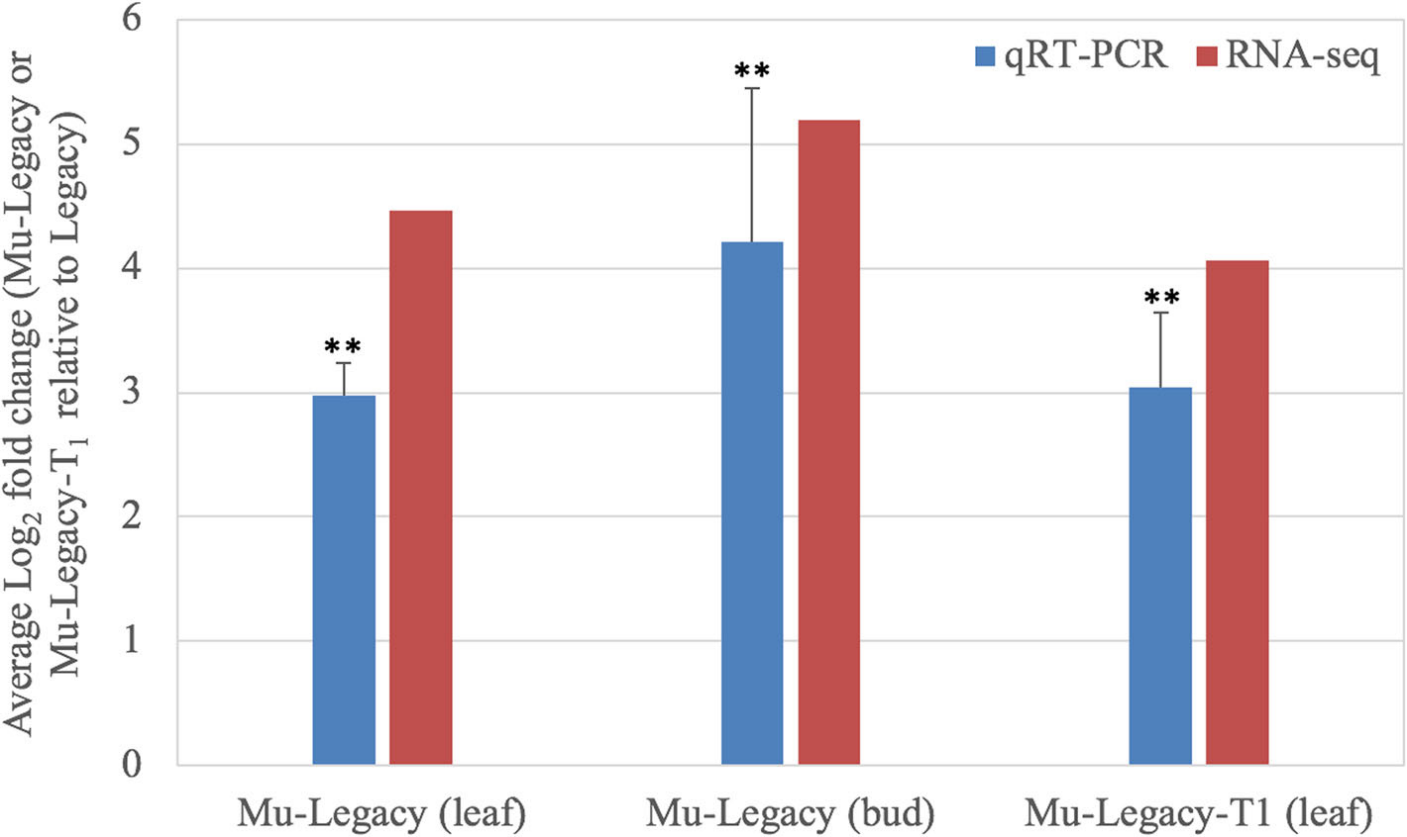
qRT-PCR analysis of *VcRR2* in Mu-Legacy and Mu-Legacy-T_1_ using E1F and E1R primers. Eukaryotic translation initiation factor 3 subunit H is the internal control. Log_2_(fold-change) in Legacy-VcDDF1-OX was calculated by -∆∆Ct = −[(Ct_GOI_ – Ct_nom_)_Mu-Legacy or Mu-Legacy-T1_ – (Ct_GOI_ – Ct_nom_)_Legacy_]. Average fold-changes ± standard deviation of three biological replicates for each of Mu-Legacy, Mu-Legacy-T_1_, and nontransgenic ‘Legacy’ plants were plotted. Significant average fold-change determined using a Student’s *t*-test is denoted. Asterisks (**) indicate *p* < 0.01. The fold change of RNA-seq is an average of the Log_2_Fold change (Mu-Legacy/Legacy) for three DE isoforms listed in Table 1

In the 47-Kbp transgene insertion region, transgenes showed overexpression; more importantly, the *VcRR2* was the only DE gene and it was upregulated in both leaves and buds (Table 1; Additional file 2: Table S1, Additional file 3: Table S2, Additional file 4: Table S3, Additional file 5: Table S4). Similar results were observed in leaves of Mu-Legacy-T_1_ (Fig. 9). Since the *VcRR2* did not show differential expression in leaves and flower buds of *VcDDF1*-Legacy [27], the upregulated expression of *VcRR2* could be responsible for the mutation of Mu-Legacy. It is likely that the CaMV 35S promoter of the *gus*A gene drove through the NOS terminator and then promoted expression of the adjacent *VcRR2* gene.

## Discussion

### T-DNA insertion upregulated expression of adjacent host genes

Regardless of transgene, a T-DNA integration is often associated with a position mutation by a random gene insertion [32]. In Mu-Legacy, the insertion broke a 354-bp retrotransposon (Fig. 5); a homologue of the retrotransposon was also identified. The role of the 354-bp retrotransposon in regulating expression of the *VcRR2* is not known in this study. In the literature, there is almost no evidence showing that a retrotransposon itself contributes to regulating plant flowering. Since the retrotransposon is adjacent to *VcRR2*, it is a genetic marker of the *VcRR2*; however, whether it has some regulatory roles in *VcRR2* expression or evolution is still to be determined.

Random T-DNA insertion is often associated with nucleotide deletion at the T-DNA borders [32], which was demonstrated in this study (Fig. 5). More interestingly, we found the constitutive 35S promoter driving through the NOS polyA terminator where a truncation occurred right after the stop code (Fig. 5), which enabled overexpression of a downstream *VcRR2* gene in Mu-Legacy (Table 1, Fig. 9). Consequently, the Mu-Legacy and its transgenic progeny showed early flowering, altered chilling requirement, and reduced plant height (Figs. 1-4).

### Roles of *VcRR2* in plant flowering

In the dicot *Arabidopsis*, *ARR2* is involved in cytokinin responses that affect a wide range of developmental processes in response to phytohormones [33–38]. In comparison to wild type *Arabidopsis*, loss-of-function mutants of *ARR2* (*arr2*) show retarded growth and development, and early flowering [34, 39]; the gain-of-function of *ARR2* promotes in vitro shoot production and leaf differentiation, and delays leaf senescence [35]. In the monocots (e.g.*,* rice and corn), two-component systems play a central role in cytokinin signaling, but the roles of *ARR2* orthologues are not clear [40–42]. B-type *RR* orthologues are identified in woody plants, for example, in black cottonwood (*Populus trichocarpa*) and peach (*Prunus persica*) [43].

Here, for the first time in woody plants (blueberry), it was found that an enhanced expression of *VcRR2* in Mu-Legacy plants, in addition to the overexpressed *VcDDF1*, resulted in reduced plant size, early flower bud initiation and flowering without chilling, and enhanced flower bud formation (Figs. 1-4, Table 2). Although the roles of the overexpressed *VcDDF1* can not be excluded in the Mu-Legacy plants, our recent studies have demonstrated that the overexpressed *VcDDF1* alone were not able to change chilling requirment in the other 48 *VcDDF1-*OX lines [26–28]. Functional analyses of *VcRR2* through overexpression, gene knock-out, and phytohormone profiling are still needed to reveal the roles of *VcRR2* in blueberry flowering.

### Flowering pathway genes and chilling-mediated flowering of blueberry plants

Comparative transcriptome analysis provides a powerful tool to study the differentially expressed genes associated with phenotypic changes in transgenic blueberry plants [20, 21, 26, 27]. In terms of flowering time regulation, overexpression of a blueberry *FLOWERING LOCUS T* (*VcFT*) (an increase of approximately 2900-fold) was unable to completely eliminate the need for chilling in blueberry for normal flowering [20]. More recently, overexpression of of the *VcSOC1K* increases blueberry productivity by promoting early and more flower bud formation through the other DE MADS-box genes [44]. In this study, flowering of nonchilled Mu-Legacy plants has demonstrated non-*VcFT*-mediated plant flowering, where early flowering and reduced shilling requirement was associated with decreased expression of *VcFD*, *VcTFL1*, *VcARP6*, and *VcDOF5.3* without the involvement of other major flowering pathway genes (Additional file 2: Table S1, Additional file 3: Table S2). Further transcriptomic comparsions of these transgenic plants will reveal the DE genes that are involved in chilling-mediated flowering in blueberries.

The transgene insertion position and *VcDDF1-*OX in Mu-Legacy caused 18 DEGs and 31 unannotated DE transcripts (Fig. 8; Table 2). Most of the 18 DEGs have not been well-studied in plants. The obvious phenotypic changes associated with a small number of DE genes in the leaf and nonchilled bud tissues of Mu-Legacy make it invaluable for studying the chilling-mediated flowering mechanism in woody plants (Fig. 6, Additional file 1: Figure S1). Further investigations on the effect of chilling on expression of flowering pathway genes in the progenies of Mu-Legacy will allow the unravelling of the mechanism of chilling-mediated flowering in woody plants.

## Conclusions

Mu-Legacy was identified from 49 *VcDDF1* transgenic events. The most obvious phenotypic change is that Mu-Legacy was able to flower under nonchilling conditions, whereas nontransgenic ‘Legacy’ and the other transgenic events could not. In addition, transgenic progenies derived from the self-pollinated seeds were also able to flower under nonchilling conditions, but none of the nontransgenic segregants in progenies derived from Mu-legacy, or transgenic progenies from another *VcDDF1* transgenic event, flowered. Since the mechanism of chilling-mediated flowering remains unknown in woody plants, Mu-Legacy and its progeny provide a unique material to study woody plant flowering. The significance of *VcRR2* in Mu-Legacy suggests that the *VcRR2*-involved cytokinin pathway likely contributes to the major differences in chilling-mediated flowering between woody and herbaceous plants. More importantly, Mu-Legacy shows increased yield potential, a decreased chilling requirement, and better winter hardiness than many low-chilling cultivars growing in southern warm winter conditions.

## Methods

### Plant materials

A southern highbush blueberry cv. ‘Legacy’ is tetraploid and needs more than 800 CU for normal flowering. Forty-nine transgenic ‘Legacy’ plants contain an overexpressed *VcDDF1* [28]. Both transgenic and nontransgenic plants were developed from in vitro cultured shoots; they were grown in the greenhouse (heated for winter) or the courtyard between two greenhouses under natural light conditions and a regular schedule of irrigation and fertilization using 0.2 g/L fertilizer (Nitrogen: Phosphorus: Potassium = 21: 7: 7).

Plant chilling was conducted in an unheated hoop house in winter under natural light conditions or in growth chambers at 4 °C with a 12-h photoperiod. The conversion of selected temperatures to chill units for highbush blueberry is based on the equation: total chill units = 0.5 × number of hours with temperatures below 2.4 °C and 9.2–12.4 °C + 1 × number of hours with temperatures 2.5–9.1 °C – 0.5 × number of hours with temperatures 16–18 °C -1 × number of hours with temperatures above 18 °C [45].

One hundred transgenic plants of a representative *VcDDF1* transgenic event (herein VcDDF1-Legacy) and two hundred plants at different ages, for each of the nontransgenic ‘Legacy’ and Mu-Legacy (a flowering mutant of transgenic legacy), were grown for phenotyping experiments under various chilling conditions, including chill units of zero, 133, 300, 500, and 850 under controlled conditions or above 850 CU in the open-air conditions in Michigan. For each treatment, three plants for *VcDDF1*-Legacy and at least five replicated plants for each of the nontransgenic ‘Legacy’ and Mu-Legacy were used.

Self-pollinated T_1_ seeds of Mu-Legacy were stored in a refrigerator for six months prior to their germination either in soil or on half strength Murashige and Skoog (1962) medium (MS) [46]. The seedlings germinated on half strength MS were micropropagated prior to being grown in the greenhouse. All T_1_ progenies were grown in the greenhouse and were not exposed to any chilling. Plant size and flowering time were documented.

### Mapping of the T-DNA insert in mu-legacy

Identification of the T-DNA insert in Mu-Legacy was conducted using the PCR method [32]. DNA samples of nontransgenic ‘Legacy’ and Mu-Legacy were obtained using a cetyltrimethylammonium bromide (CTAB) method. Both *Hin*dIII-digested and *Eco*RI-digested DNA samples were ligated to adapters and then used for PCR using adapter primer AP1 and T-DNA border primers according to O’Malley et al. (2007) [32]. Eight primers to cover an approximately 200 bp region from each end of the T-DNA were designed based on the sequence of the T-DNA. Primer and adapter sequences used in this study are included in Additional file 7: Table S5. Target PCR products were recovered from gel, purified and ligated to a pCR 2.1-TOPO vector (Invitrogen, Carlsbad, CA, USA) for sequencing.

The identified 1279 bp sequence of the blueberry genome at the T-DNA insertion position was used to search the blueberry EST database (http://www.vaccinium.org). A 781 bp expressed sequence tag (EST) (CV091265) from nonacclimated floral buds, which has a 199 bp overlap with the 1279 bp sequence, was used as a reference to design the primers (i.e.*,* E1F & E2R, E2F & E2R, and H2F & H2R) (Additional file 7: Table S5) for further extension of the 1279 bp sequence. PCR products were ligated to a pCR 2.1-TOPO vector (Invitrogen) for Sanger sequencing.

### DNA sequencing and genome assembly

Total DNA was isolated from 200 mg young leaf tissues for each of the Mu-Legacy and Mu-Legacy-T_1_ (a selected T_1_ progeny of Mu-Legacy) sample, using a CTAB method [47]. The samples were purified using DNeasy Mini Kit (Qiagen, Valencia, CA, USA). The integrity of the DNA samples was assessed using electrophoresis. All samples were sequenced for 150-bp pair end reads with about 40-fold blueberry genome coverage using the Illumina HiSeq4000 platform at the Research Technology Support Facility at Michigan State University (East Lansing, MI, USA).

For each of the Mu-Legacy and Mu-Legacy-T_1_, SOAPdenovo2 and ABySS/1.9.0 (k = 64) were used to assemble genome sequences using the resources at the High Performance Computing Center at Michigan State University.

### RNA preparation and sequencing

Nontransgenic ‘Legacy’, Mu-Legacy, *VcDDF1*-Legacy (named II7 in our previous report [27, 28])were used for RNA sequencing (RNA-seq). Three three-year old plants of each genotype were used for collecting leaf and flower bud tissues. Nonchilled flower buds (30 to 50 buds) were collected in November before the plants were exposed to an unheated hoop house for chilling. Fully chilled flower buds, 30–50, were harvested at the end of January from courtyard-chilled plants. Young leaf tissues were collected in February. All the collected tissues were frozen in liquid nitrogen immediately and stored at − 80 **°**C.

Total RNA was isolated from 0.5 g tissues for each sample, using a CTAB method [47]. The samples were purified using the RNeasy Mini Kit (Qiagen, Valencia, CA, USA). The integrity of the RNA samples was assessed using electrophoresis. All samples had an RNA quality score above 8.0 prior to the submission for sequencing (100-bp pair end reads) using the Illumina HiSeq2500 platform at the Research Technology Support Facility at Michigan State University (East Lansing, MI, USA). The FastQC program (www.bioinformatics.babraham.ac.uk/projects/fastqc/) was used to assess the quality of sequencing reads for the per base quality scores ranging from 30 to 40.

### Differential expression analysis

The RNA-seq reads of three biological replicates for each of the nontransgenic ‘Legacy’ Mu-Legacy, and *VcDDF1*-Legacy were analyzed. Each biological replicate was sequenced twice. The paired reads, two sets for each biological replicate, were aligned to the transcriptome reference Reftrinity [20] and the abundance of each of a single read was estimated using the Trinity command “align_and_estimate_abundance.pl”. The Trinity command “run_DE_analysis.pl --method edgeR” was used to conduct differential expression analysis [48]. The differentially expressed (DE) genes or isoforms with the false discovery rate (FDR) values below 0.05 (*p-*value < 0.001) were used for further analyses of the flowering genes of blueberry. Most of the analyses were performed using the resources at the High Performance Computing Center at Michigan State University. Sequence alignment and phylogenetic tree analyses were conducted using CLC Sequence Viewer 7.

### Quantitative reverse transcriptase polymerase chain reaction (qRT-PCR) of DE transcripts

The RNA samples used for RNA-sequencing, including samples of three biological replicates for each of nontransgenic ‘Legacy’ and Mu-Legacy, were used for cDNA preparation. Reverse transcription of RNA to cDNA was performed using SuperScript II reverse transcriptase (Invitrogen, Carlsbad, CA, USA). The resulting cDNA of one microgram of RNA was diluted (volume 1: 4) in water and 1 μl/sample (25 ng) was used for PCR reactions. PCR primers that cover the *VcRR2* region (E1F & E1R) were used. Eukaryotic translation initiation factor 3 subunit H was the internal control (1). QRT-PCR was performed in triplicate on an Agilent Technologies Stratagene Mx3005P (Agilent Technologies, Santa Clara, CA) using the SYBR Green system (Life Technologies, Carlsbad, CA). In each 25 μl reaction mixture, 25 ng cDNA, 200 nM primers and 12.5 μl of 2x SYBR Green master mix were included. The reaction conditions for all primer pairs were 95 °C for 10 min, 40 cycles of 30 s at 95 °C, 60 s at 60 °C, and 60 s at 72 °C, followed by one cycle of 60 s at 95 °C, 30 s at 55 °C, and 30 s at 95 °C. The specificity of the amplification reaction for each primer pair was determined by the melting curve. Transcript levels within samples were normalized to the eukaryotic translation initiation factor 3 subunit H. Log_2_(Fold changes) were calculated using - ∆∆Ct = −[(Ct_GOI_ – Ct_nom_)_Mu-Legacy or Mu_Legacy_T1_– (Ct_GOI_ – Ct_nom_)_Legacy_] for each transgenic Mu-Legacy versus a nontransgenic ‘Legacy’ sample (*n* = 3) [49]. In addition, regular RT-PCR was also used to verify the specificity of the primers prior to qRT-PCR analysis. The reaction conditions using 50 ng cDNA per reaction for all primer pairs were 94 °C for 2 min, 35 cycles of 45 s at 94 °C, 60 s at 60 °C, and 60 s at 72 °C, with a final 10 min extension at 72 °C. RT-PCR products were separated on 1.0% agarose gel containing ethidium bromide, visualized, and photographed under UV light.

### Statistical analysis

Comparisons of quantitive traits (e.g., the number of flower buds, plant height, and the number of fruit clusters) between Mu-Legacy and nontransgenic ‘Legacy’ plants were made using a Student’s *t*-test or one-way ANOVA Test in R3.3.1.

## Additional files

Additional file 1: **Figure S1.** Representative flowering patterns of two-year old transgenic T_1_ progenies of Mu-Legacy (a-k) and a nontransgenic progeny (l) under nonchilling conditions. (DOCX 230 kb)

Additional file 2: **Table S1.** Differentially expressed (DE) genes in nonchilled flower buds of Mu-Legacy (compared to nontransgenic ‘Legacy’). LogCPM: log_2_(counts per million). FDR: False discovery rate. (XLSX 62 kb)

Additional file 3: **Table S2.** Differentially expressed (DE) genes in leaves of Mu-Legacy (compared to nontransgenic ‘Legacy’). LogCPM: log_2_(counts per million). FDR: False discovery rate. (XLSX 31 kb)

Additional file 4: **Table S3.** Differentially expressed (DE) genes in nonchilled flower buds of Mu-Legacy (compared to VcDDF1-Legacy). LogCPM: log_2_(counts per million). FDR: False discovery rate. (XLSX 243 kb)

Additional file 5: **Table S4.** Differentially expressed (DE) genes in the leaves of Mu-Legacy (compared to VcDDF1-Legacy). LogCPM: log_2_(counts per million). FDR: False discovery rate (XLSX 281 kb)

Additional file 6: DNA sequences at the insertion position of Mu-Legacy. (DOCX 26 kb)

Additional file 7: **Table S5.** Primers used in this study. (DOCX 18 kb)

## Abbreviations

ABA: Abscisic acid
bp: Base pairs
CORs: Cold-regulated genes
CPM: Counts per million
CTAB: Cetyltrimethylammonium bromide
CU: Chill units
DE: Differentially expressed
DEGs: Differentially expressed genes
ESTs: Expressed sequence tags
FDR: False discovery rate
GA: Gibberellic acid
Gbp: Giga base pairs
GO: Gene ontology
Kbp: Kilobase pair
MR: Million reads
MS: Murashige and Skoog medium
Mu-Legacy: Mutant ‘Legacy’
qRT-PCR: Quantitative reverse transcriptase polymerase chain reaction
T-DNA: Tranfer DNA
*VcDDF1*_OX: *VcDDF1* overexpression
