# Supplementary material for: An invaluable transgenic blueberry for studying chilling-induced flowering in woody plants
Source: BMC Plant Biol. 2018 Nov 1;18:265. doi: 10.1186/s12870-018-1494-z (PMC6211425; doi:10.1186/s12870-018-1494-z)
Supplement: Supplementary file 1 — Figure S1. Representative flowering patterns of two-year old transgenic T1 progenies of Mu-Legacy (a-k) and a nontransgenic progeny (l) under nonchilling conditions. (DOCX 230 kb) [file 12870_2018_1494_MOESM1_ESM.docx]

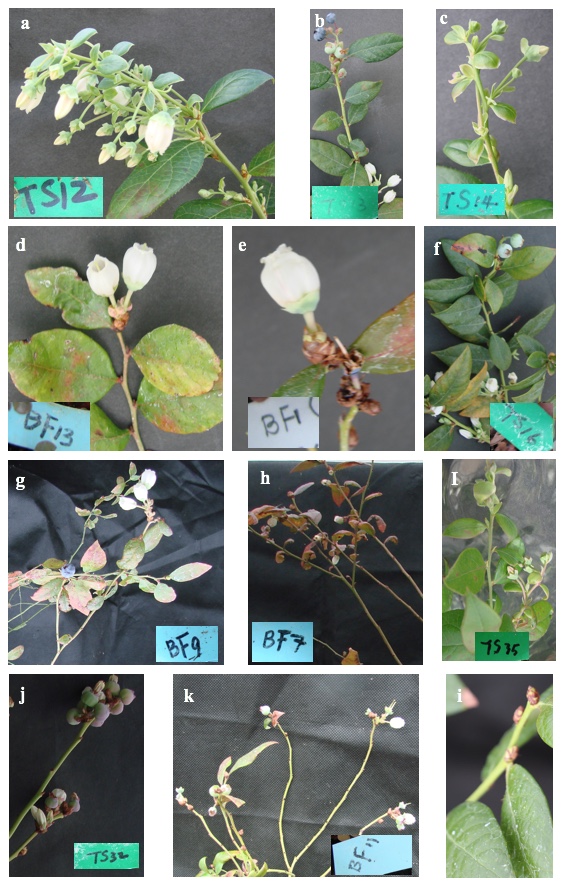


**Fig. S1** Representative flowering patterns of two-year old transgenic T_1_ progenies of Mu-Legacy (a-k) and a non-transgenic progeny (l) under non-chilling conditions.
