# Supplementary material for: An invaluable transgenic blueberry for studying chilling-induced flowering in woody plants
Source: BMC Plant Biol. 2018 Nov 1;18:265. doi: 10.1186/s12870-018-1494-z (PMC6211425; doi:10.1186/s12870-018-1494-z)
Supplement: Supplementary file 6 — DNA sequences at the insertion position of Mu-Legacy. (DOCX 26 kb) [file 12870_2018_1494_MOESM6_ESM.docx]

**Supplemental information 1** DNA sequences at the insertion position of Mu-Legacy

5'-

AGAGAGAGAGAGAGAGAGAGAGAGAGGGGCTTCACTCGCTGGTGGAAACTAAAGAAGCCAAAGAGGCGATGCCAGGCTTATACATCGCCAATCGGTTCAGATTTGAAGAGCAATGGCATCTATCTGCCATGGGATAATTTTGAAGCCAAATTAAGGAGAAACAGGGCACCTCCAATCTTGAACAGAGAGAGAGCTCAAAACTAAAGGTAACGACGCATGGCTTGTTGAAAAAAGAACCTCCAATCACAACCGAGTTTATGGTGTAATCTTCCCTCGCCTCTGTTTTACAATCTGGATCAGCTGATTGATGTAAGGTACAGGAGACGCTGGTTGAGCTGATGAGCTTGCTTCTGCTGGGCCTTTTCCTTTGTTGTTGCCGGTAATTCCACTTTCAGCTAAATCTTCATCCATGGCTTCTCTTGGATAGGTTTGAGCAGTTCTGGATTCATTCACTCTCGTAATCTCTGGTATACTTGAGCTAATCGTTCTACCTTTCTAGTTGTGTAGAAGGTAGATAATAGCAAAACCTACTAATCTTATGATACTGAACAAAAAATGGAAACCAATAATAAGAACAAATAACTTGATACCTAATTCTTCGAGAAAATACTTCGAGTTTCTTTTAATGGAATACTTCAGATATTCATCTTTTTCTTCGAGTAGAATATTGCTGGTATCTCTAAATTATAAATACTTCAGGTATTTTTTTAGTGACAAATAAAATATTTCAGGTATTAGGGGCACACTACTTTCAGTGCGACACCCTTTTTGGTTAGAGTTCCGTGTTGATAACGTGTTATAAGTGCAAGACAAATAATAAAGAAACTTATGTAAAGTAAAACAAATAATAATGAACACAATATGTATATGAAATAAGTTCTTCTATGACTATTGAGATCGTCTTCTAGACTCATCATAGTGTTCTCTCTCTAGATTCTCTAATGAATAAAACTGATAACGGAAGGCTCTACCAGATGAAGCTCCAGGGACATATCCTTGCAAATAAGTTAGGATGTGGGACTAGTGTGTAATTTACCCCACTGTTTGTAGCCGCCCACCGTATTCAATCAATTCATATCAGTATGTATGCAAAGTCTCCTCCTTGATATGTTGTTCAACTTGTTCTTTGTAAATTTCTCTTGCTACAGGCGTTGCTCTTGAATTGCCCTATCCCAGTATTCTCTTTTGAGTCCTTTCTAATCACCTACCCGAATATTTAGACCGGCTTTTCATCAGTTGCCGATCTTCATTTTCAGTTGTGGACACATACAAGAAAAGAGGTATGTCGTAAGTCCTGACACCCTCATATCGTAAGTCATGTAATTTTTTAAATCTTATTTAAATGTTCTCTTTTTATGTTACAAGCCACTGAATTTCTAGTTTTGTCTCGCATTAGAAAATTGATAAACAAATTGTTAAAAATTGAAATGTCGACGTCTCAAATACTTTATTTTGTTCCTTTTTTGGTTTTTGAATGTAGAAAAAAAAAATTTACCTTGCTTTTGGATTTACAGATCAGTTTCATATCAAATCAAACTTATTTTCTAGCAACAGTTATAAAGTTTGGTAAATAGGCCCAAAAAGGAAGGAACCAAAAAAATGAAACAAATGCTAAACCATCCACATGAGGTCCAAAAGAATGCCACCAATGTGAAGCGTAATACTAATAATCTGCTTATCTGAATCGAAAAATACATAAAAGACATCCATCATATGATCATAAGTATCCACATGAATATTGAGTATCAAATTTCTTTCACAAAGGTTTTTGAATTACGTTGGTTGTGCGGCAGGCCCCCTAAGAACTCAACTACAACCCAAGATTATGGATGGCTATGATAGTGGAGAAGTAACAAACTAAACATATGCGAAATCAAAATGGAGAAACCAAAACCAAAACCAAAAGCATAAACAAATAAGAGAGACACCAAGATTTACGTGGTTCGGCTTACAGATGTAAAACTACTCCACGGGGCAAGCAAACGAGAGAAGATTCCACTATGTGATGAGAACATGCTCCGAAGAGCTACAATGGTGACAAATAAACAAAGCTCTCACAAGAGACACTCACCAACCCCTATTACAAGTTCTCTCACCAAAATCCAATTAGAATCTCTTACCAAAACTGATTCTCTCTCATCAAGTCGCATAAAACCGTTAGGGTTTTTAACCAAGAGACGCAGGAAACAAAGATCTTCCCAAGCCCACCAAGGAGGAGCAACCAAAGAACCATGAAGATCTAACCAAGAACCCTAATTTTATATGAGACCTCCTAGGGGCCTCTCAAAGGGATGCATTCAAGCAGTCCAAACCCTAATGGAGAGAGCAAGGCTATATTTTTAGCCACAGAACCCTTCCTAAAGTCAATACTTGCCCAAATAGATTTCCTTGCACCCCAAGACATAGCCAACAAGGAAATAATTCTTTAGGAAGTCCAAGACCAATTCAAAACACAACAAAATCGGTTAAAACGTGCAAAAACGGACCCGGTGGTCCGGATCAAGACCCGATCGTCCGGACCAAGCATGCTGCAAGCTTCCATTGTATACCAGATTCATTGGTTCGGACCAAGGGTACCAAGGTCCGGACCAAACATGCTGCGAGCTGCAATTTTGTCCAAAATTGCCCAAAATTGCTTGTTCCGGACCAAAGAAGCCTTGGTCCAGACCAACCATGCTGCGAACACCAAAATCGAGCCAAAATTCCAACAATTTGTAATAGCTCCAAGCAACATGATATAGTTTTTGTCTTATGTATTTCATTTTCCATCATGTTCTTGTAGGTTTACTAGTCTAATATCTAATTAGCTCACTGCATTTCAATCTCGTATTTGATGCACAACTCTTGAAAATTGGGAGCTTCAAGTGAACCGAAAAAAAATGGGTTTTCAGGGGTCCAAACAGAGTCCTTCTCAGCAAATTTTGGTGACCCTAAAAAGCATCAAAACTGTGTCATTTTGATCCACAGCGTGTTGCACTGCAGCAAGCACGGTAACGAGTTTAGCAATACCACCAGTACCAAGAGGGCACAATTTCATCTTTTGTGCTATTTATTGTCCAATGGAAATGCAATCCATTTACAAAACAAAAATATCCTATACTCAGATTTTACTCAAATTAAGAGATAAAAATTTCCCCTCAAATTTAGCATTGTGCCATTATGAAGCTTGTACTCCAATTGATGAGCATATTTGAGTTTTGAGTGCCTGATATGCTTTAGAACACTTTGCACATGGTGCACAGGATCGCGTGGTGCGGGGATTCGGTTGTTCATAGCGAAAATGCAAGGCTCGAATTTTATCCAAACAATGGACGGTTTGGATTTACACGGGGCTCAAATTAAATCCGAGCCATGCATTTTTACAATGGACGGCAAGATGCCCACATGACACAGTGCCGTGCACCAAGTGCACAGTAGGAACTCCTCTTCCGCTTCAGAACATATGAAATTTTTACTCAAGTTTTGCAAAGCTATTCTTCCCCCTCAAACTTATAAACCTGCCACTACAAAAATAAACTTTAATTTTTCCCTCCAAAAATACACAATACGAAGCTTTCATTTGGTATGGAGCACTCCAATTTGGCAAATGCAAATCTAGCCACAAAATTGAATAAACACTTCACAAATCTAGCCTCCCAAAAATTGAGTAAGAAGTTAATCCCCATTGATATCGGTAAAAATTTGTGTAAAACTCAATTTGGTATAAATTTGTTTTTCTTTTAGAGGGTGAAGGGGGCTCTAACTCAAAATTTGGGTTTTGAGCCAAATTTGATATAAAGAGAGTTACAAAAAGGAAAAAAAGGGAGAGAGTGGATATGCTCTGAAGATTTTTTAGTATGCACAAACAAAAATGAAATTTTTACTCAAAAAATGCAAGGCTATTTTTTCCCTTAAAATTAAAATCATGCCACTAAAAAAATTAGGTATGAAGCTTGAACTCAGGTTTTGAGTAAAAATTTGACTAAGGGGCTGGAAATATTCCAGTATGGACAAATATAGGGGGCAAGTAACCAAATGGTGGTCATAGCGGTCAATTGACTCACCTAGGTGGTGCATAGGCCACCTAGCCTCACACGTGGAAATGACTATGGGAGTGATTGGAGGGATTAGTCTAGTTCATCAGACATTCTTCGTTACCCAAAAAAATAGGGAGCAAGTGTTTTAGAAAATTTGTGAAAAATGTACCCGGTGACTGGTCTTTCCCTTAACGGAAACATAGATCCTGCCATGGACGAGTCGAACTCCAGGCAATTTATTTGTGGAGGGGCACTCTTCGTTACCCAAAATTATTGTTTGATTTTGAATTTTGGGTAACGATTGAAGGGAGAAATGAGGGTAATGATTGAAAATATGGAGAATAATTGGGGAGACGTAGAGAGAGAAATAATAGCAGTAGAGACACAACTCATAAAAAGATCAATCCATTTAGAATCAAAGTTGAAATAAATGAGAGTATCTTTAAGAAAGGTCCAAGAAATTGTCATAAGCTTTTTCAAGATCAATTTTAAAAATAAAGGCCACCTTTTTAACCTTTCCTGGTATTAAGGGTATGGAGGATTTCCCAAGTAATAAGGATGTTGTCATTTGTGGACCTATCAGGAATGAAAATTTTGGTGATAACTTTATATCACAGTACAAAGACTTATGGGTCTAAACATCTTGATGTTGTCGAGGTTATCGAACTTGGGGATAAGAGCAATGAGGGTTTTGTTAATGTCCGCAGGAACGGAGTTAGAATTAAAACAATCCTTAATAAATTGACAAACTGCAGAGTAAGCAGTATTCCAACACTTGTGGTAAAATATTGCGCGGATGCCATCAGGGCCAAGGGCGTCCGAGCTGCAGAGATGGGTCTATCAAGTCGCCTAAtggcaggatatattgtggtgtaaacaaattgacgcttagacaacttaataacacattgcggacgtttttaatgtactgaattaacgccgaattaattcgggggatctggattttagtactggattttggttttaggaattagaaattttattgatagaagtattttacaaatacaaatacatactaagggtttcttatatgctcaacacatgagcgaaaccctataggaaccctaattcccttatctgggaactactcacacattattatggagaaactcgagcttgtcgatcgactctagctagaggatcgatccgaaccccagagtcccgctcagaagaactcgtcaagaaggcgatagaaggcgatgcgctgcgaatcgggagcggcgataccgtaaagcacgaggaagcggtcagcccattcgccgccaagctcttcagcaatatcacgggtagccaacgctatgtcctgatagcggtccgccacacccagccggccacagtcgatgaatccagaaaagcggccattttccaccatgatattcggcaagcaggcatcgccatgtgtcacgacgagatcctcgccgtcgggcatgcgcgccttgagcctggcgaacagttcggctggcgcgagcccctgatgctcttcgtccagatcatcctgatcgacaagaccggcttccatccgagtacgtgctcgctcgatgcgatgtttcgcttggtggtcgaatgggcaggtagccggatcaagcgtatgcagccgccgcattgcatcagccatgatggatactttctcggcaggagcaaggtgagatgacaggagatcctgccccggcacttcgcccaatagcagccagtcccttcccgcttcagtgacaacgtcgagcacagctgcgcaaggaacgcccgtcgtggccagccacgatagccgcgctgcctcgtcctggagttcattcagggcaccggacaggtcggtcttgacaaaaagaaccgggcgcccctgcgctgacagccggaacacggcggcatcagagcagccgattgtctgttgtgcccagtcatagccgaatagcctctccacccaagcggccggagaacctgcgtgcaatccatcttgttcaatccccatggtcgatcgacagatctgcgaaagctcgagagagatagatttgtagagagagactggtgatttcagcgtgtcctctccaaatgaaatgaacttccttatatagaggaaggtcttgcgaaggatagtgggattgtgcgtcatcccttacgtcagtggagatatcacatcaatccacttgctttgaagacgtggttggaacgtcttctttttccacgatgctcctcgtgggtgggggtccatctttgggaccactgtcggcagaggcatcttgaacgatagcctttcctttatcgcaatgatggcatttgtaggtgccaccttccttttctactgtccttttgatgaagtgacagatagctgggcaatggaatccgaggaggtttcccgatattaccctttgttgaaaagtctcaatagccctttggtcttctgagactgtatctttgatattcttggagtagacgagagtgtcgtgctccaccatgttatcacatcaatccacttgctttgaagacgtggttggaacgtcttctttttccacgatgctcctcgtgggtgggggtccatctttgggaccactgtcggcagaggcatcttgaacgatagcctttcctttatcgcaatgatggcatttgtaggtgccaccttccttttctactgtccttttgatgaagtgacagatagctgggcaatggaatccgaggaggtttcccgatattaccctttgttgaaaagtctcaatagccctttggtcttctgagactgtatctttgatattcttggagtagacgagagtgtcgtgctccaccatgttggcaagctgctctagccaatacgcaaaccgcctctccccgcgcgttggccgattcattaatgcagctggcacgacaggtttcccgactggaaagcgggcagtgagcgcaacgcaattaatgtgagttagctcactcattaggcaccccaggctttacactttatgcttccggctcgtatgttgtgtggaattgtgagcggataacaatttcacacaggaaacagctatgaccatgattacgaattcgagctcggtacccggggatcctctagagtcgacctgcaggcatgcaagcttTGAGACTTTTCAACAAAGGGTAATATCCGGAAACCTCCTCGGATTCCATTGCCCAGCTATCTGTCACTTTATTGTGAAGATAGTGGAAAAGGAAGGTGGCTCCTACAAATGCCATCATTGCGATAAAGGAAAGGCCATCGTTGAAGATGCCTCTGCCGACAGTGGTCCCAAAGATGGACCCCCACCCACGAGGAGCATCGTGGAAAAAGAAGACGTTCCAACCACGTCTTCAAAGCAAGTGGATTGATGTGATATCTCCACTGACGTAAGGGATGACGCACAATCCCACTATCCTTCGCAAGACCCTTCCTCTATATAAGGAAGTTCATTTCATTTGGAGAGAACAgcgcgagtctcccactattatacggtcccgagaagagtgagttagacgtgcgcttgtacacgagtctcccactattatacgatccggagaagagttaaaacgtgcgcagctttatcctgcaagcaaaaaggtttgcttacgaacacgcgaatcgtcgatggaatataactctagtccacattccggtgcattttttccaatcgggtcagactcaggatccaaatctccttcattagaatacgtcgaaggttcgaccagccgaggctcaccgcaatcggacgaggagctgatgataacactggcttcgagccagccgaagaagcgagccgggaggaagaagttcaaagaaacaaggcacccaatctacagaggagtgaggaggaggaacaacggcaggtgggtgtgcgagctgcgggagccgaacaagaagtcgaggctatggctcgggacgtacccgacggcggagatggctgctcgggctcacgatgtagctgcattggcgctgaaaagtgagtcgtcgaaggcctgtttgaatttcgcagactcggtgtggcggttgcccgtgccggtatcgaaggaggcagaacatataaggagggcagcggccgaggcagctgaaatgttccggcaacagccaccaaacgatggtggcagcgtgaacgtcgtctcctctgatggagatggttcaggtgggaatgggagtgaggtttttgacatggatgaactgcttttgaatatggcggaagggccgttactgtctccttcgccttcttactcgggtcgtcggttcagctgggatgatgttggaagtgatgttgaggtctcgttgtggagttagattttagatgaacgaaattcaaccatgtataaactcgattgagccaaatagctgctttgtttatgcagagaaatatgtataatttggtgtaacgcgatgttaaaacaatgaaattGATCTGGATTTTAGTACTGGATTTTGGTTTTAGGAATTAGAAATTTTATTGATAGAAGTATTTTACAAATACAAATACATACTAAGGGTTTCTTATATGCTCAACACATGAGCGAAACCCTATAGGAACCCTAATTCCCTTATCTGGGAACTACTCACACATTATTATGGAGAAaagcttggcactggccgtcgttttacaacgtcgtgactgggaaaaccctggcgttacccaacttaatcgccttgcagcacatccccctttcgccagctggcgtaatagcgaagaggcccgcaccgatcgcccttcccaacagttgcgcagcctgaatggcgaatgctagagcagcttgagcttggatcagattgtcgtttcccgccttcagtttagcttcatggagtcaaagattcaaatagaggacctaacagaactcgccgtaaagactggcgaacagttcatacagagtctcttacgactcaatgacaagaagaaaatcttcgtcaacatggtggagcacgacacacttgtctactccaaaaatatcaaagatacagtctcagaagaccaaagggcaattgagacttttcaacaaagggtaatatccggaaacctcctcggattccattgcccagctatctgtcactttattgtgaagatagtggaaaaggaaggtggctcctacaaatgccatcattgcgataaaggaaaggccatcgttgaagatgcctctgccgacagtggtcccaaagatggacccccacccacgaggagcatcgtggaaaaagaagacgttccaaccacgtcttcaaagcaagtggattgatgtgatatctccactgacgtaagggatgacgcacaatcccactatccttcgcaagacccttcctctatataaggaagttcatttcatttggagagaacacgggggactcttgaccatggtagatctgagggtaaatttctagtttttctccttcattttcttggttaggacccttttctctttttatttttttgagctttgatctttctttaaactgatctattttttaattgattggttatggtgtaaatattacatagctttaactgataatctgattactttatttcgtgtgtctatgatgatgatgatagttacagaaccgacgactcgtccgtcctgtagaaaccccaacccgtgaaatcaaaaaactcgacggcctgtgggcattcagtctggatcgcgaaaactgtggaattgatcagcgttggtgggaaagcgcgttacaagaaagccgggcaattgctgtgccaggcagttttaacgatcagttcgccgatgcagatattcgtaattatgcgggcaacgtctggtatcagcgcgaagtctttataccgaaaggttgggcaggccagcgtatcgtgctgcgtttcgatgcggtcactcattacggcaaagtgtgggtcaataatcaggaagtgatggagcatcagggcggctatacgccatttgaagccgatgtcacgccgtatgttattgccgggaaaagtgtacgtatcaccgtttgtgtgaacaacgaactgaactggcagactatcccgccgggaatggtgattaccgacgaaaacggcaagaaaaagcagtcttacttccatgatttctttaactatgccggaatccatcgcagcgtaatgctctacaccacgccgaacacctgggtggacgatatcaccgtggtgacgcatgtcgcgcaagactgtaaccacgcgtctgttgactggcaggtggtggccaatggtgatgtcagcgttgaactgcgtgatgcggatcaacaggtggttgcaactggacaaggcactagcgggactttgcaagtggtgaatccgcacctctggcaaccgggtgaaggttatctctatgaactcgaagtcacagccaaaagccagacagagtctgatatctacccgcttcgcgtcggcatccggtcagtggcagtgaagggccaacagttcctgattaaccacaaaccgttctactttactggctttggtcgtcatgaagatgcggacttacgtggcaaaggattcgataacgtgctgatggtgcacgaccacgcattaatggactggattggggccaactcctaccgtacctcgcattacccttacgctgaagagatgctcgactgggcagatgaacatggcatcgtggtgattgatgaaactgctgctgtcggctttcagctgtctttaggcattggtttcgaagcgggcaacaagccgaaagaactgtacagcgaagaggcagtcaacggggaaactcagcaagcgcacttacaggcgattaaagagctgatagcgcgtgacaaaaaccacccaagcgtggtgatgtggagtattgccaacgaaccggatacccgtccgcaaggtgcacgggaatatttcgcgccactggcggaagcaacgcgtaaactcgacccgacgcgtccgatcacctgcgtcaatgtaatgttctgcgacgctcacaccgataccatcagcgatctctttgatgtgctgtgcctgaaccgttattacggatggtatgtccaaagcggcgatttggaaacggcagagaaggtactggaaaaagaacttctggcctggcaggagaaactgcatcagccgattatcatcaccgaatacggcgtggatacgttagccgggctgcactcaatgtacaccgacatgtggagtgaagagtatcagtgtgcatggctggatatgtatcaccgcgtctttgatcgcgtcagcgccgtcgtcggtgaacaggtatggaatttcgccgattttgcgacctcgcaaggcatattgcgcgttggcggtaacaagaaagggatcttcactcgcgaccgcaaaccgaagtcggcggcttttctgctgcaaaaacgctggactggcatgaacttcggtgaaaaaccgcagcagggaggcaaacaagctagccaccaccaccaccaccacgtgtgaattacaggtgaccagctcgaatttccccgatcgttcaaacatttggcaataaagtttcttaagattgaatcctgttgccggtcttgcgatgattatcatataatttctgttgaattacgttaagcatgtaataattaacatgtaatgcatgacgttatttatgagatgggtttttatgattagagtcccgcaattatacatttaatacgcgatagaaaacaaaatatagcgcgcaaactaggataaattatcgcgcgcggtgtcatctatgttactagatcgggaattaaactatcagtgtttgacaggatatattggcgggtaaacACATGGAATAACAAAGTGAAATAATTCTGGACAAGGTCCTTCAGTCTAAGAGGATCATCAACCCATTCATTGTTCTCATTCTTAAGCATGTTAATGTTAAAGTTTTCGGTTCATTCATTGAAGTTTGTGGGGGCATGCTAAAGTTTTCGGAATGATGGCTTTACCCCATTTTGTCAATTGTGGTGATTAAGGAAAATAAAGAATCAGGGACGTTGAAGGCATAGTCTCCTTTTCTTGAAACTAGAAGTCGAGCACATGCGATGTGTGTGCAAGTCGAACTTTGACAGAAAAAATATTTCTTGAAAATGCGTGCCCAAGTTAAACTTAAAATATATATGCATGTGGGTATTTTTTTTTTTAACAGACCAAGTTTCATGAAATATGGATTGATTTTAGTTTGGGTCATGCCCTAAAACACAAAATGAAGGATTGGGCAGGGACAGAAGTGAAATAGTTCAAAAGTATAGGGGCAGGTCAGTGGTTTCGTCTGCCATTTTTTGTTCACCAAAACGAAGAGCGGCGGTGGGGAAATGGGGATGCATATCTCTCCTCCTTCCTTCGTTTCCTCTTCGTTGTTTTCTTCTCCTTATTTTCCGCACAAAGACTGTCCAACTGGGAATGGAAACCCTCTTTCCCTTTGCTTCGTCGAGAAGCAGCACCTGACATCAGTACAATTGATAAATATCTCATCGATACACTGTATCTGTATTCTGACATCGCCGTTTCTCGATTTTGTTTGCCCTTCCCTGATTTTGTGGCCGCTTTGCTCTTGCCATGGAAGTACACACCCgtagctgctagcacagtgatggcgagactcgtactcttctggtactggtaaaccctaattacctcttctttgtccaaaacacagaaggactattatcaagaattcagtactcttttctggttagtaggaatagcttaacatcccgcttatgagatagtttatgctaaaataattttatagttgtatactcgtgttggaatcagcaagataaatgtagcgtacatctttcctttcctatactcgtgtgaacggggttcgattattggttagtttcaaagggcgcattcaatattaaacaatggagagcaaggagaaagactatctccttctgtgtcattggctccgaaacaagggattcccaacagctagaagtcgtcttcgtctaacactttcacatacttacctagatgggattggtgtcatttggtttacaattctgcaacctaaaaccatatgtaactctactgctgcatataatgcttatttactaactccttaatgtttgtatttgtcatgtctgaatcactgtaatttggatacgcagaagttcagactatatttgaagagcttaagtggagtaactcaacgaggtgggattcccaattcttattttggccctcttgaacaaaatagaaatcatggttctctaggaagattcgatatccaagctttggctgcttctggtcaaattcctctgcaaacattgctagctctgcatgctgaatttctaggtcgaccaactggtacggtagctaaaagtatatccatgaattgggtcttaagactttctttatgtttttctttctctagacaatagctactacctcgctgattatggttgtaccttctcccactcaatgtcatttccggatgcttatttgtttagattccaaaactctgtacgaagaaaagagggaagaattatatgaaaacttgaagaccaatgaatctattggatgggaagttgatgtcatagatccaaggagctcccagctaaaatgctgaagaaggtagtagataagtgtttttctttatttcatcttgttttgatgcagcaccattataagataattcgtcctatttgattttgaccacgaataaaacaagcctgatgaaatttcacaggagtttacaatgggtcttgtcaaatgggtgctgaacacgggagttcttttgtgttcgtttcattttctggttaggttcaattagtttacgtgtgactgtagatttttagccacatattcctaagagtatccatttatatgctaatgatatcggcaaagccttaaatttggttcctacttgtagccatatgagattaatgaaatgcaattctccaatgacaggatttcaaaattgaatacagatgggtgcttccaaagcaatctgggaaaagagacattcagatggatgtctggggatgataagtcaattggctggtagccctctgtggaaggattcaaaactgtaaagtagacttgggagcaataaaatgaggtctgttcaacatgctaaagctagaattgattccataagcttttctatagaaaagtatatgcaattgttgcaactgctatgtaaaatgagaaagtaagagcaacattcagtcatcaattcattcatacagaccaaataagtgaagttcatgatgagtgtgcttgaaaaatgaaaaaggtgaaattccaaataaattgcgaatgggacaagcttattactattgctttggcttgcatttttgttttgcttcttgctgttgttttatctcaacattctgcggttttctttgatctcaacatctttaagatgtctttatgctagtttgctttgatctggaaagttagcacagatcacagaagataattcagccgaagagcattaGTTGAAGCCTCAACAAATTTGAATCTTCCAATTGTGGTTCATTTATTTCATCGAAGGTGGATAGCATATTGTCATTATATTAGTTTACATTGATATGTATTTTTATGGTACTATTTTTAAGATTGTCAACCTTGACCTTGAGAGCAATTTTGGTGGTGCTTAATGTGCATGTCTACCTCATGGTTTAGTGTGACTATATTATTCATGGAAATAAATGATCTAGAAGTTTACAATAGATAGATGAAACAGTTGTTTTTTCACCTCTTCCATTGTGAATTCTGTCATTTTGAAACCAATGACTGAAAATTGACTTCTCGCAAGCTTCAGTGTTCCGGTATAACCATCTTGTACGTATTCTGCAGTACCTCAGTGAGGTACAAGTCCGACTTCTCATGTCCAAAAACAACTTGAATCGACTTCATCAATGTTCACCAATGCTTGATGGTCGTGTGTCTGACATCGATAGAAGTTGTTGGTTTGTAGTCCTCCACTCGTTCTCCATTCTGTCCCTCTGACGCTCTCTTTGCAAAAAAATCTCGAGTTTGTTGTTGATTAAGCGCACTTTGAGGTTAGTTGCTTTATTTGAGTTTGAAACTGAGTCCTTGGTTTTCCTATTTTGATTTCATTTTAGAAACATTTTTCCCTGTAGATCTAATATTTAGCCATTAGTATTACTATCATTGGCATTTTTGGGTTATTGTCTCCAATTTGTAGGCACATGTCAGAAGGATTAGCACCCGTTCAATTTTGTAATCCTCTAACTATGATCCCTTGGATGATGCAGGAGTCAatgagatagtttatgctaaaataattttatagttgtatactcgtgttggaatcagcaagataaatgtagcgtacatctttcctttcctatactcgtgtgaacggggttcgattattggttagtttcaaagggcgcattcaatattaaacaatggagagcaaggagaaagactatctccttctgtgtcattggctccgaaacaagggattcccaacagctagaagtcgtcttcgtctaacactttcacatacttacctagatgggattggtgtcatttggtttacaattctgcaacctaaaaccatatgtaactctactgctgcatataatgcttatttactaactccttaatgtttgtatttgtcatgtctgaatcactgtaatttggatacgcagaagttcagactatatttgaagagcttaagtggagtaactcaacgaggtgggattcccaattcttattttggccctcttgaacaaaatagaaatcatggttctctaggaagattcgatatccaagctttggctgcttctggtcaaattcctctgcaaacattgctagctctgcatgctgaatttctaggtcgaccaactggtacggtagctaaaagtatatccatgaattgggtcttaagactttctttatgtttttctttctctagacaatagctactacctcgctgattatggttgtaccttctcccactcaatgtcatttccggatgcttatttgtttagattccaaaactctgtacgaagaaaagagggaagaattatatgaaaacttgaagaccaatgaatctattggatgggaagttgatgtcatagatccaaggagctcccagctaaaatgctgaagaaggtagtagataagtgtttttctttatttcatcttgttttgatgcagcaccattataagataattcgtcctatttgattttgaccacgaataaaacaagcctgatgaaatttcacaggagtttacaatgggtcttgtcaaatgggtgctgaacacgggagttcttttgtgttcgtttcattttctggttaggttcaattagtttacgtgtgactgtagatttttagccacatattcctaagagtatccatttatatgctaatgatatcggcaaagccttaaatttggttcctacttgtagccatatgagattaatgaaatgcaattctccaatgacaggatttcaaaattgaatacagatgggtgcttccaaagcaatctgggaaaagagacattcagatggatgtctggggatgataagtcaattggctggtagccctctgtggaaggattcaaaactgtaaagtagacttgggagcaataaaatgaggtctgttcaacatgctaaagctagaattgattccataagcttttctatagaaaagtatatgcaattgttgcaactgctatgtaaaatgagaaagtaagagcaacattcagtcatcaattcattcatacagaccaaataagtgaagttcatgatgagtgtgcttgaaaaatgaaaaaggtgaaattccaaataaattgcgaatgggacaagcttattactattgctttggcttgcatttttgttttgcttcttgctgttgttttatctcaacattctgcggttttctttgatctcaacatctttaagatgtctttatgctagtttgctttgatctggaaagttagcacagatcacagaagataattcagccgaagagcattaGTTGAAGCCTCAACAAATTTGAATCTTCCAATTGTGGTTCATTTATTTCATCGAAGGTGGATAGCATATTGTCATTATATTAGTTTACATTGATATGTATTTTTATGGTACTATTTTTAAGATTGTCAACCTTGACCTTGAGAGCAATTTTGGTGGTGCTTAATGTGCATGTCTACCTCATGGTTTAGTGTGACTATATTATTCATGGAAATAAATGATCTAGAAGTTTACAATAGATAGATGAAACAGTTGTTTTTTCACCTCTTCCATTGTGAATTCTGTCATTTTGAAACCAATGACTGAAAATTGACTTCTCGCAAGCTTCAGTGTTCCGGTATAACCATCTTGTACGTATTCTGCAGTACCTCAGTGAGGTACAAGTCCGACTTCTCATGTCCAAAAACAACTTGAATCGACTTCATCAATGTTCACCAATGCTTGATGGTCGTGTGTCTGACATCGATAGAAGTTGTTGGTTTGTAGTCCTCCACTCGTTCTCCATTCTGTCCCTCTGACGCTCTCTTTGCAAAAAAATCTCGAGTTTGTTGTTGATTAAGCGCACTTTGAGGTTAGTTGCTTTATTTGAGTTTGAAACTGAGTCCTTGGTTTTCCTATTTTGATTTCATTTTAGAAACATTTTTCCCCGGATCTTGATTACTGAAGTTGGTCGCTTCCAATCTAGTACAGCTTCTACCTTGCTGTCGTCAACTGCAATACCATCCTTAGACACCACGTGTCCCAAGAATTTAACTTACTCTAGCCAGAACTCGCATTTACTAGCTTTAGCATAAACTGGTTGTCTCTTAGTACTTGCAGTGCTGCCAGAAGGTGTCCTTCGTGCTCTATTCTGTTGGCTGAGTATATCAAGATATCGTCAATGAACACTACCACAAATCTATCTAGATAGGGTTGGAATATCTTGTTCATGAGACACATGATTACTGTCGAAGCATTTGTCAGTCCAAAAGGCATGACCACGAACTCGTAGTGTCTGTATCTTGTCCTGAATGCGGTCTTCGAGATGTCCTCGTCTCGTACTCTCAACTAATGATACCCTGATCTTAGATCAATTTTCGAGAAACAAGTCGCTCCTCTCAACTGATCAAATAGGTCATCAATTCTAGGCAGTGGATAACGGTTCTTAATCGTGACTTGGTTCAGCTGCCTATAGTCAATGCACATCCGTAGGGTTCCTTCTTTCTTCTTCACGAATAGTGCAGGAGCTCCCCATGGTGAAGTACTTGGTCGGATGTATCCTTTTCCAACAACTCTTGTAGCTGGGTCTACAGTTCCTTCAGTTCTGTTGGTGCCATTCTGTATGGGGCTATAGAAATCGGGGCGGTTCCAGGTTGAAGCTCTATGGTAAAATCGATCTCTCTTCGTGGCGGTAATCCAGGTAAATCGTCAGGAAACACGTCAGCATACTCGCAGACGATGTGTGGCAGTCCTAACTCTTCTCGATTAAATTCTTCCAACTGGAGACTAGCTAGCCATCCGAACAGTTGGTTCTGCCATTTCGAGCGTCTAGTCGCCGAATTCACAGCCTATCTATCTCCCTTAAAACGGAAACAAGTCCCATCAAGGGTAAAGGTTGTTACTATCTTTTGATGACAATCAATGACTGCTCGATGAGTTAACAGCCAATGCAATCCCATAATTAATTCGAAGTCCGCCATCTCGATCACGCGTAAGTCGCAAGTCAGTCGAATATTACTCACTTCTAACTCGTAACCTCTACAGATTAGATTAACTGGTAAACTACCGCTTAAAGGTGATGTTATTGTCATCTGGTTCTTTAGGGGTTCAGTGTCTAATCCTAGTGCAGATACGCATGCAGCTGATATAAACGAATGTGATGCTCCAGTGTCAAATAAAGCTTGTACGCAGGTCCTGAATAACAGTAGCGTACCTTGAATCATAGAAGGGTCCTGCTCCTGGATCTGATCCTCGGCATGTAGAGCGTACAGACATCCCTTCCCGTTCTGAAGTGCCTTGACCTGCTGTTTCCCATTCCAGTTGTTCTGCTGAGCTGGTCTAGCATTCTGTTGCATAGGCTATTTTGCCGAAGTTCTGAAGCGGCTGCGGCTGCTGCTGCTGCAACTGTTGATTCCCATAGGTCAATCCATCCTTCCTCGGGTGAGGGCAGTTGCGACTGTAATGTCCATAGCCCGTACAGTTGTAACACCTGACCGGCTGGTTGTTATAGTTGGAATTCTGTGGCGGCGGTCCTTGTCTGATTTCATTTCTTCTTCAAGGCTGGTTGTTAACTTGGTTAGGTTTGGCTGGGTAGGGCCCACGATTGTTGTTGTTCTGCACAGTCCTAATAGGTCCTTCTGAATCGCTTCCTTGTCGTTTAGCCCAGAAATCTACTTCCTCTCTCTCCATCCTTAGGGCACATTTCACTGCCTCAACATAGGTAGTATAACCGATTCCCATAACCGTCTTGCGAGTCGAGCCAAGTCCCCATTCAGACTTTCGGTCCTTGTCCTCCTCAGCTGGGATAGTGGCAAGGCCATAATGGTACAGGTCCTTAAATTTAGCGACATACTGCGTCACTGTCATTAAACCTTGCTGCAGGAAGTACGACATGGTTTACTATAGTACCGAATTTTAATTGATACAAACCATAGACTTAAAAGTCAACTTACAACCTAGGTTATACATATTACAACCAAATTGAAACTTCCTTACTGCTAGTTCAATCCCAAACATCCTTACATTACAACTTATTTAAAAACCTAACTAATGCTAGGTTATAGCCATCTCCATCATCCACTTAACTTCCTTAGAACATCCACGGGAAGTATGCTCTCAACATGCTTTCCCTTTCCACGGTCACCTCGGTTATGCCATAATACCTCCAGGTCACTCTCCACCTTTTGTCTCTGTGTATGATCCCAGAGGGGTCAATGAACTCTTCCTTCCTAACTGGCAGCTCTGAGTAAGTTCGGCTGTCGAAAAAGTCTAGGTAAAGTGAGATAATCCGATGTTCGTCTGCTGGGCGAACACGGGATACTCGGAGATATAGGATCTTGTTGGATAGGCGCGCAGAATATCCATGAGGTAGGTCAACAGTGTAGTATCGATTTACACAAGGCTTAGTGATCTGCACAGGACCCAAGAAATATCGCTGATTGTGCTCCTTCCAGAAACAAGGAAACACTGGGGCTATCCCTATGTAGAATCATTCACCAAGCTACAGGTTAACTAGGTGTCCGTCAATAGGGGCCCAGTTTGGTAGTCTCATGCCAACCGATATAGGTGGTAGGCTAAAGGGGTCATCAACCTCACTTTCAGGATCAGATTCGGGTTCAGGTGGTGGGTCAACAGGATTCTGTACTAGGTGCAGTGCGTTGGCTTCAGCCTCATAGTCAGCTAGCCATCCATGCATGTCAAAATTTAGGTGTCCCACCTCATCTTCATCATCATAATCATGAAAATACATCATAGGATTAGCATCATCCTCCTCTTCATCATTGTTCTCAAATTCAGGGTTAGACAGATACCCTCCATCTTCCTCAGAGTCATCTTCCGGTTCATTAGGATAATCTTCTGGTTCCTCAGGGTCACTTTCTGGCTACTCAGGGTCATTTTCCATATCAGGGTCATCAGAATCGATGTCGGGATCAAAGAGCATTGGTGGAAATTTGCCATGTCGGCGGAGATAGTTGGTTATTAGTGGAAAATTCTCGTGGACACTCAATAGATACCCTACCCCGACCTTAAACTAGCAATTCTAGAACAATATTGTAGGTGGAAGGTAAATAGTAAAATCATGCATTTTCAAATATCAGAGTTAAAACAAATCGAAGTGCAGCTTCACAAAATCAGTGTCTCGTTTCATTTCTAGAATTAGAGTCCAGTGTCAATATCTCGTATCAATTTCTCATAACGGCATTCTTTTTCAAATTTCAACCATGTCAACATCTCACATCTCTCGAAAGTTATTGGCCCGTTCAACCACCCCTTTCTAACGGTCACCAACCTTTCCGTTCGAAGTCCCGGCTATCCATAACACTGACTACCCGCTGGTTATGAAGTCCGTTCACCCCGACTCACACACGACGTAGTCTCGGCTACCCATATCACTGGATCCAACAACCTATTTATGAAGTTCGTTCTCTACATGTTGTACTAAGTGAGCTTTGACTGCCAAATTCACCCCGCGGAATTTGAGGTGGAACTCCAAACAGCACTACTGTATAGTTCCGTTGAGCTTTGACCCCCAGTTCACCCCGTGGAACTGAGGCGGGACTTCAACAGCATTATCGTCTCAACTTTCATCACATCATCATTTTCATTCTCATCTCTATCTCTTCTTGTGAAAACAATCGTACCATCTCGGAAAACGTGCACGTGCCATAATTTCATAAAAATCCTCCGTTTAAAACATTTCTGATAAATGTGTTACTCAAAGACAAAAATTCTTTCTTCCGAAAATTTAAAACAAAACATTAAGGGTTATGCTTGCTTTTGGATTTGTTGGCGTGTTTTAAGACTCGTTTGTCGAGTCGCCACTTGGTTTGGGCTAAACCAAGAAAGAGCGTTACTGGAAACTCTAGTAGTTTGAATCCGTTGGCGTGTTTTAAGATCCGTTCTTCGAATCGCCACTTGGTTTGGGCTAAACCAAGAAAGTGCTTGACTAGACATTCTAGTCATGGATTCTATTTTTCAGCCTAAACAAATTTAAAATATTTATTTAGTAATTCCGGGAAATCTCGTTTCATTTGGAAAAGCTCTTTAGTTCAGAAAATCAGTCGGACATTATAACTGCGTAAATTTGAAAAGTCGATTTTCATTCGGGCATTATAACTACATAATTTGGTTTTCCAAAAGTGCATTGGGCATTATAACGGTGCATTTTGTTAAGTAAATCTCAAATCGTGCATAGTAACGACTAAGTGTGTTTTCAAAGTCTAATTTTTCATTAAACCACTAATCATCCAACATAATCTCGTTTAAACATAATTAAGCACATTTAAGAAACCAAAACTCAAAACCCAAAATCCAAACCAAACACAACCATGTTTTTCTCATTTTTACTCTTCAATTGCTATTGTATTTTGTTACTTTCTTATTATTTTTAATTTCAAAAGGTGAAATTTTTTAACAATAATCCATTTATTTTTGAAGTGGGACAATATTTTTTTGGACGACAAAAAGTGTCAACTGAGACTATAAATTGGGGACGAAGGGAGTAGACGAGAAAGGAGAGTCTTTTTCTTACCAAATCTCACTATTTTCTGTGAGATACATCTCACTGTTCTTTTGTGAGAATGGGAAAGAGCCAATTAAAATTTTTAGTTTTTGTACTTTCCTTCGATTTTTACCAAAATACTCAATCAAAATTAGTGATTTGGTTAGAAACCAAACCCCTTTTTCCTTGTTTTCCTTTTTGGCCTCTCACAAAGTCCGTCGCAAATTGAAGTACATAGGAGGCTAGGGAAAATGTGAGAAAAGAAAATGAAAGGAAAATTACATTGGGCTGGGCTTAAATAATTGGATTTTTCTCTTCTTTTCCTTTCTTTCTTTAAGGGGGTGTTCCACAGATTTGGGGGAGGTGCTGTGCACTTGGGTGCTTTGCACCGTGCAGTGCACCTCTCAGCTATCCGATTGTGTTTTGGAGGGTCCCAATGTGTTCGAAAATTTTAATTTAATTTTGATTTATTTTTACCGATAAAAAATCAAAACACATCCGGACCATCCAAAACACAATCGGATGGCTGAGGGATGCACAACATCGTGCAAAGCACCTTGGTGCACAGCACCCACCCCTTGTCCCAAATTCCATTCCGGGCTGGTCCTCACTTTTTGGGGTTTAAAGAAGAAAAAAATTGAGTTTATGGATTTCTTGAGAAAAAAAAAAAGTTTGGGTTCTTAGGTTTTTTTGGTTTGGGCTCAATCCATTGGGTGTTTCGGCTCGGCCCATAGTTGTATGTTTGGGGTTTCGCACTTGGCCCATTTGGGTTTTTTTTTTTCTATTCGTCATTCCTAATCGATTACATGGGGGGAGGGGGGAATGCGACTCGATCCCGGGATGTAATGTGGGGCATTCAAGAGCTCTACCGCATGAGCTACCCACCACTTGCGCCCTTTTGGTGTTCCATCCAATATAATATTGGATGCTCAATGTACATTTATCGAGTTTAATAAAATTTCTCTTTTGTCGATAAAAAAAAAAAAAAAAACACAATACAAACTGAATGCTCGTATGCTATAACCAGCTACCAGCTAACTAAAAAGAACATTTAGAACTCAGAATACTAAATTACTAACCCTAACCAAGTTTTACACTTTTAGTCTTTTTACTGATTTTGTTATTTGTTTTTATGATATTGTCGACCCAACAAAACAAAATTTTTGATTTTATCATTATTGACATGAACTATTTCAACTATACTATTTTTGTGGAACTTAATAAGTCAAATACCATGTGTACGTGAGCAAGAATGTGAGCGGAAACTAGAGCTCTTGCAAGAACCCTTGGGAACACATTCATTTTTAAATTCCTGTTCGCAAGGATTGGGAGCCAAGGGAGAATCCTATTAGGATTATGTCACACGGGATTGTAGTCGCTCTTGAATTGATCCACAATTTCAAACTTCCAACATAGCCAGCTGCCCACGTAATCCTTGATGACATTTACAAATTTCCATGTATTTTATAATATCGAGACACTTTTTATACATTAACCGTTGGCACATGGTGAATCGTCTTGACAGCGGCGGCGGTCAGATGGTGGTGGTTTCCGGCGAACGGGAGGCGTGGTGGTGGTCGGCGATGGTAGGTGCCCTCTCTCTCTCTTCGAGTTTTGACTGAAGTATGGTGGATGGGAAATGGGGAAGAAGGAGAAGATTTCTACGGTTTTTTTTATTTTTAAAAGCAACAGGACGTCGTTTTGGGGCTGTTGTCCAATTTGTTGTCTAATTTGTTGTGTATGTAGCATTTCCAAACTGGAACACCCCAAAATATGAAGCCTTTTAAGCATCTAAATTAACCATCAATAACCAAGAACGGAAGGAGGGAGAAAGATTTGATGTTGATGTTGATGTTAATGTTGTTGCAGGAGCCGCAGATCCTTGCCTGTTTGGTTTGGAGGGGGAAAGTTTTTTTTGGGAATCAAGATCTTCTTATGTGATAATCCCGTGTGATTCCTCATGTGACGTAAAATGTAAACAATTACGGATCCATTTAACAATTCATACAGTTTCTTGTATTTTATAATTCTCAATACGTTTAACGTCTATGACAAAAATCAAGTTGATCGGATATTGATATCCATTTAACATATCTACNAGTCACAAGAGAAATAGATAGTCAAACTCTGCCTGCTCATTTCTCTTCCCACAATTATGATTCAACCAGGCTTTTTATTGATCTCTGATCAATTNAAATTTTTGCATATACGTTGAAGACATCGAGGCCTACTACATGAACGGTTCAGATCATGAAATAAGATTACGATAAGTCCCATTTTGCGTCACATGAGGAATTGTGATTCTGATCGAATATGTTATCCGCATAACCACCGGGGTGGTAAACGGACCTAGAAGACTAGAAGTTACTGTATAAGGAAGGCATAGTTGGGTGAGAAAAGAAAATACATGATTAGAGTTTCTCTCTAAATCACTCTTTTGGATGTATAATTTGATAAATAAATGAAAAGAACATTGTCAAAAGAATTTTGATTGGTTTCTTCCCTTTTGATAAATAAATGAAAAGAAAAGTGTCACACCTTTTTTTTTTGGATGGAGAGATAAAAATTTCATTAATCTACCAATCAAAATACAGCCACCTACAAAAAAATAGAAAAAAACAGAAATGGAAAAAATAGAAAAGGAAAAGGAAGAAACAAGAAACCGTTGGTGCATGCGCCGCCACTGTCAACCGATGTCGGAAGCCCAAATCGAGGGGGTCTCGATCTGGCTCCCCCCTGATCCAAAAATCGGGCGTGGTTGCCCATACTGGAACGGAGAGGATCGTTCGCCACAAGGAGTCTCCGAACGCCAAGAAAATGCAACCATAGGAGATTGGAGNAGTGTGGGAGGAAAATTAACTGGTATGGGTGGTTAATTTGGATGAATTTATCTTCCTACTCTTGCAGACTAGAGGTGTGAATGTGTGATTGTTTCATAGGGTAAATTTTTTATTTTTTATTTATAATTTGGGACTTAAAAAGTATGAGACAGCCTTCATTGGGAATCCACATACCAAAGTGTGCTCTCTCTTTAAATGCCACAAGGTCACTAAAAAGGATGTTAAGTGGATAACCCCGCTGCCTCTGTCATATCCTTCAATTTACACTCAGAACTAATCCATTCCCTGACATTAGAGTTGGCTACATTCACAACCTACAAGGAAGATAGATTAAATCCTATTCTTTTCTCCTCTGTCCTCAAGTTCCACTTAAATTTGGTTAATTCATTGAAAAAATGACAGAAAATAATATTACACCAAGCTTAAATAATACTAGTTACAAACAGTGTCACCTTCACTAATTTTATAATTTTTTTTAAGATTTCGACATTTATCTAACGATGTAGGACGAGATATTAAGGGCTATCACTTATTGAAATAATAGGAAACCAGTATAAACCATCTCCAAGCAAATCATTACTTGGAAACCAGGACTATAAAAAAGTAAAAAAAAATAAAATCTTATCAAAACTAAATTAAGAACGTGTAAAACAGGCAACACGAATAAGAGTTTTTTTAATACTTACCGTAATTAAAAATTATTACCGAAATAATATTTTCATCAGTGATAGTCCTTAATATCTCGTCCTACATCATCTAAAGAAGACTTTTGACTTTAATGTTACTGATCATACATAACTTGTGTTTCGCACTACACAATGCTAGACTTCCAACACAGTTGGTCGTCCACGCGTTGATGACGTGGTGACCACGAATAGTCTATGTGATATAAATACGCCGTACTACTCAAGTAGTATCTAAAAGAATCACTTAAATATCACAATTCAAAAAAAAAAAAGTTGAACATTACAAGATTTTAAAAAGTATCAATTGATCTTTCTGTTTTAGCAGCAAACGTAATAATAATATGCTGATACAATCAATCTTATTCTTCATCTCATGTTTAATAGATGCAATTGATATTCTATTCTTCTATTCTCCACTTGTTAGTGATATAATTGTGCGAAAGTAAAACTTAATCAATTTCACTTTTGAAAAGAAAAAAAAAAACTGTGGAAACAACTGTATCGGGAATAGAATATATAAACTTTAATTTTTTTTTATGGGTACTGATATAGAACGGGCCCTTATAATTTGCATATTTTTTTAGGACTAAAGGGGCAGCACCTTGAGCTTTGTTTTTTTTTTTAAAGAAGGCAACCTTGAGCTTTGTTTAGCACAGGGCAACACTGATTCTATTGTTTGTTTTTATAATATTGTTGACCCAAAAAAAACTTTTGATTTTTGTTTTATGACCACACACTGTTTCAGTCATATTACTTTTTGTGCATCTCAAGTAAAAAATAATGCATGCGCGAGCAGCGCGAGCATGAGTGGAAGCTAGAGCCCGGAAAGGACCCTTCAAAAATATATTAATGACTTGTTGGATGCCGGATTGATTTTGGGTGGATGTAGAAGGAGGCTGGATTTGAGGGTGAGCCCACCTGACCATTGGCGGAGCCACTACAAAACACTGGATTTTAGGGTTTTTTTTTTTTTGTCTATTCTTATAGAAAATGTATTAGAAATTTCACCGGTACATCATATTATTTGTACAAAAGATTAATTATTGTTTTTGGGGTCACAACTATATGGTGTTATAACATCATTGTCCTTGATGTGGGACTTGAGACTAAATATATGAGTTTTATCTAACTATGAGATGATGAAGGCCGAAAAAAAAGGGTCATGGAGAAGGTTTTTAGGGCACCATGTGGAGATACCCCACAACCATCGAATAACTAATTACTATAAACAAACTTCAATTAACTGAATTTTTGCCTCAAAACTATTAGGTTTTTATGGATAATCATTATATTACCATAGAATGCAAGTCTTTTTTTCCTTACGATATACATCAACAAGGGATAGTAAAAAAATTAGTGTGCTAACAACTAAAGAGAGTTGGAAATCTCTCAACTAACTCGAACCACAGACCTCCCCTTGGAATTCAAAGGTTACACCCAAATAGGCTGAGGCACATTTGTCCAAGAGAGGCTCCTTTCAAATGAAAATCAACTAATCAAGGCATGTTAGATTCTTAAAAAAGAGTATTTTTTTTTACACTACAAAGTACTGGGCGTTTATGGTCTAATTGTGCCCTCACTGGTGTGATTTTCTGGCTACGCCACTGCACCTGACCACCGTATATGAAGGGTGGATTGTATATCCAGTGTTTAGATTTTATATTTTTATGTTTGAGAAGAGTGATTAGGAAGGTGGATATGATGGAACAAGTTGTCAAAAGACTTTATTGCCCCCACACGAGTTATTTCATCTCAAATTTGTTTTAATCACTATTAATTACAATAAAATAACATTTACCACGAATTGTGTTATTAAATATTTTATTTTATGAGAGAATGTTTAAATATTATTTTTATTACAAAAAATATTTTTCCCAAAAAATTTAAAAATGAAATTGTGTGTTTTTTAATGTCTATAAAATTTATTATATATGTGAATGTGTATATAGAGTGAAGTGCACATTTATATTTATAATGTGATTTTTTTTTACGAAAAAATTGAACAATAAAATAATGGGACTTTACAGTTGAAAAAAATAATTGAAAAGTTATATAATGTGATTTTGATTTGTTTACCGGTTTTGATATCCAATTGAGCTGATTTTTATTCAAGTTTGTTTAATGTTTTGAACGTCCATGTGAAAAATTAGCTTAATCGGATATCCATAGGGTTTTTTCTAACGGCTCAGAACACACAAGTGAGGGGTACAATTAATGGGAATTTTTTTGGGCAAAAATAATTGAAAATTTATATAATGTTAATTTTTCTTTTTTTTAAGAATACACCCCTACAAGGCTAACAAAATTAGAAAATACTTCAAGCGATTTTCGGTATCCAATTGAGCAAATTTTTATTCAAATATGTTTATTTAATATTTTGAACGTCCATGTGAAAAATCAGCTCAATCAGAAATCAATAGGAGTGTTTTCTAACGGCTCAGTATTCCAAAATGCTGTTTAAGCTATATTCGTAGCCTACCCAGCTCNGCTATGCAGGAGCATCCCTTTGTGACTCACGCNGAGCTAGGGCATCCTGATTTATCAATTCACTCTCATTAGTCTGNCCTTATTCATGGTCAAACCCAGCTCGGCACAGGGCCTATGGTTTCNNTATGTGGTGCCGAGTTATTCACTCTCTCATGGTGATCGATTAGGGCACGTGCTATGCCTTCCATTAGGGTAATCACGTTTTACCCACTCCATAATGTAAATGGATATAGGGTGCAAGTTTTATTTGGACCAACCGAACCATAATTTTTCTCAAATTTTTTGATCAATCACACCATTTGAGAATAGCAATTGCTCCCTAAATGAAAAAACGTTGCATCTTAGGTTCTCTCAACTTGTCAAAATGAGGTGGTGGTGTGGTACATAATTTGTTGCACCGTAACTGAATTAGGTTTGCACCTTGTATGAACCAGCATTGCTCCTTACCTCAAACTCATTGCACTAGGTTAGGCATTTGCTTCTTGAGTGAATTAACATAACATCATTTGAGAATAACAATTGCTCCTTAAACGAAAAAACATTGCATCTTGAATGAATTAATATCCATTTGTCTAAAAAAAAAAAAAAACCATTAGCTCTTTAAATGAAAAAATGTTGCACCTTGACTGAATTAACATGGCACCATTTGAGAATAACATTGCACCCGGATGGGAACGAATGGGTTCTTTTAATCCAACTTGTTTAATTTGGTAAAAATAATTATTGCTTATTTGATTTAAAAATATTGCACAAAAAAAANANACATTGCTTCTCACACCATTTGAGAATAACATTGCACTTGAATGAGAACGAATGGGATGCCAGGGTGGCCTAAATCCAACTTTTTATGTTAGAAAAAATAACCATTGCTCTTTAAATGAAACAATGTTGCACCTTGAGTGAAGTAACTTAGCACTAGGGGTGAGCACGGGTCGGGTTGGGTCGGGGTTGGCACCCACCCGACACCCGACCCGATTCTATCGGGTTAGAGTTTTGTGAACCCGCAACCGACCGACTACACTAGAAAAACCCGTCCGAAAATCATCATCGGATTGTCGGGTCGGGTCGGGCGGGTTCATCGGTTTGGGCCAGGAAAGAAAAAAAAAAAACGGCGAGATCACCCGGAACCCGAAATTTATTTATTTTAAAACCCGAACCCGACCCTATAAAACATTAGAATCCGCCCGAACCACTGCGGGTCGGGTTGGGTCGGACGGGTTGGGCGGGTTGAACGGGTCCTTGCTCACCCCTACTTAGCACTATTTGAGAACAACTTTGCACCCAAATGAGGAAAACAACATTTGCTCCTTGTATTACCAAATTTTACACCTTACGTGAAAAAAATGGCACCATTTGACAATAACATTGAACCGGAATAAGGACCTAATTTGATGGGATAGATTATCTATGAAAATAGTTGGAATCCTTGGGCTCCCTTACTCTGCTTACTTGCTCTCTCATCTCTAGGTTGTTGCCTTCACTTAGACAATATTACAAACACCTTGCATGCAAGAAAGTAAGAAAGAAAAGACACGACATTGTAGTTGTCTCTAATAGAACAAATTAAATTCAAATAGAGGGGATGTTACATGAAGCATTTTTCGAATCAAATATATAAATAAATTCCATCTTTCCGGCATGAAATTATTCCAGTAAGTAACCGATAATAGGTTCTACTAATGACAACATCAACTTGAGGGCATTCCGCAGCCATTGCATATGGTTGTGATCCTAAATACAATAAAGTGCAGTAGAAATAGTGGAGAAGAAGCATATAAACACAGGATATAATTGTTCACAGATTGATCCATTCTTCGTCATCATTAAAAATTGGACTCAGAAACATGAAATCGGTAAAGTTCATAACGTAAGACCCAATTGAAAAGATTAATTTATAGGGTACCAATATTTTTCATCAAAGTATCTTTTATGCTTTCTCAGGAACTAGAGAAGGAAAATTTCCACAGATCTGAAGATGACTGTTGGATGATGGGTCTACACTCTCCACCTTTTTCAACGAACGCCGTTGGGCTTGCCGCCGGTGATGGTGGTGTTCGTCGGGCGCGGTAGTGGTGGCGTCACTCTTCTATCTCCCCCTCCGGAAGACTGGAACGAAGAGAAAGGGGAGAAATATGGGAAGGGGAATGAGAGAGGAAAAATGGAGAGGGTATTGTGGGCATTTCATCTATAAAACACCATGTATGGGTAATTTTTTTTCCTTGTCATGCTTTGGGCATTTGTGAAACCATGCCCATGTTTTTTGGCATAATTTGCCCCAACTACCATGTATTGGTCATTTTCCTATTATTTTGAGAAGGGGGGGGGGGGAAGTGGAAGATGAGGAACAATGATGGGAAATAAAGTAGAGAGAGGGGGGAAGGTGGAAGATGAGGAACAATGAAGGGAAATAAAGTAGGTGATGATGAGAAACAATGATTGGGAAATAAAGTAGGGGTGTTAGAGTAATTCTAGTAGGGCATTTAACAGAGTTTGGCCGTCCGTCCACGTGTTCGACATGGCGTTCACAGCGAATCTTGTTGTCGTATTGGATAAAGACGCTCAAGTCTCGGCTGTCTTCCTCCCTCGCCACCTTCTCCTTCACTCGCTCCGAATCTCACTCTCACCATTATATAATACTGAAAAAAACTTGGCACCTTTTTCTCCTCTCCATTTGTAACTCTCTCTCTCCCCATCTAGGGTTCTCCAGATTCCGCTTTACATGTAAGCTCTGCCGATGCTTTGATTCTGTTTTTTGATCTGCTTTCTGAGAATGTGCTGATTTTGTTCTTTGATCTGCTTTTCCGAGAATGTGTTTCTGAATATACCGCAGCTGAATCGGAATCGAGAATCATTGCTTATGATTTTACATGATTTGTTGTTTTTCTGTCTGTTAGATCTCCTCGTCGTTTTGGGTGAGTACAAGTGATGTCGAAAGCAGGAGCCTTTGATCTCGCCACCGGCGTCGGAGGAAAGATCGAAAAAGATGATATTCTCTCCGCCGTCGAGCAGTATATATCTCTCTCTCCCCCCCCTCGCTCTACAATTTCTCTCTCTCTCTTTCTTTTTTTTTTTTTTTGCTTCGTTTGGCTTGCAGGCCATGTTTGATTGGATGGAGTAAACAATAAGATCGGGAATGTGGAATTTTCTAATATTCAAACGTTGTAAAATGACATGATTTATTTTCGAAATCCGCCTTCTGAGTAAAATCCAGCCAATCAAAGTGGACCTTATGAGACACTCCATTATTTAAAAACCTAGTACTGTACGAATGTTATCTACCTTCTCAGCTGTCAATTACCGTTCAGAAACAAAAAAAAAAAAAGAGGCTATCTACTGCAGAAACAAAACATTATCGTAGATCTGCTTTCCACAGTTAATTACATCAACGCTGATGTTGAAACTATTATTAATAAAGAAAACTACTCCTTCCTTCTGTCCCCGATTGTTGGTCCATTTGGAGAGCATCATCATTGTTTTCTATTGTACTGTAGTAAATAATTTGAAGAAAAATGCTATTAACAGCCTAGTGTACTGTTGTTAAGGTTTAAAAAGTGTGAAATATGTGAAAAGTATATAAAAAGTGTGTGACAGTGTATTGGAAATTGGAAAGTGTAAAAAGCGCAGTTGGAAGTGTAAAAAGCCGACGGGGCTGTCAATCGGACCCGTTTGGGATACAAAACGGCAAAAAGAGAGCCATAATTGAGGACAGGGAGAGTATCGATTTACAAAGTGAGATAGCCAAATTGCACCTATTGTGCTAACAAATGTTAAATTGATTTGGTTTCTGAGTTTATGGTGGGAAGAGAAATTACTTGTTCGTACTCATTTTTTGGTAATTAGTGTGTCAAAAAACAGTCTACTTTGTTTTCGTATAAATTGGACCCATTTATACTAAAGGACTGTGCTGCTCAATTGTTAGTGCTGGCCTTAAATTTAGTGTAAAGGCGGTTTGGAATTCTTTCCCCTTTTCTCTTCAGCTTCTACTATGATCAATTAGATTGTGAATGCAATTAGTCTTATCTGTTGCGTACATAAAAGAATGACATTCTTTTTTTTTTCCTGCATGCTGATAGTGGCATTATCATGGTTAATGCTTTTGTAAATATTTCATATTTCCTATTTATGTACTTTGTCTACTTGTCAAAAAAATATTTATATACTTAGTCTATGTAATGGTTCAGGTATGAGAAGTATCACTCTTACTATGGAGGAGAAGAGAAAGAGAGGAAGGATAACTATACTGACATGGTACTAAATTTCATTGGACTTCCTTTACATAAACTACTCGTCATTATATTATACTCTTAAAATGCATATTCTTTCGAGCAGATATATTTTGTGGTCTACGGATTACTAGAAAACAATAGTCTTACATGATGGAGTTACGGAAACTATAATTGTCCATGATCCGCCTGGTCGTCTAACTATCTACTAGACTACCTGTATGAGTGATATGCAATTTATGACCTCTGAATGCAACATTGTGTCGGCTTTGGAGTACAATTTCGGCCCTTTTAAAAAATTCAGATATCCATTGGACTCCTGAACTGATTAGATGATTCTCAGTCACAACTTTCCAACTGTGAGTGCATCCGCTTATCATTGTTTATGTTGAAGCATGAAATCAATACAACAGTTGATGTGATGCACCCACTCCAAATAAGTTCTATTTTTATCTCAAATTAATTACTTGGATGAAGAGGTAGCTGGATTAACGACTGGCATTGTGGACAATGTGGAGACTATAAACTTGTCTGTGATGAGGTTTTCAGGTGAATAAATACTATGATCTTGTTACTAGCTTCTACGAATACGGATGGGGAGAGTCATTCCATTTTGCGTCCAGGTTAATATTGGTCCCCTTTCTTTTTTTCTCACTGGTCGATTTCACACTTTAGCTACTTGATGCATTACCTGCATACAGAGGTGCCTTCGTAACTTTTTGTGATGAAATAAGTATCATGGTTCATGCAGATGGAAAGGGGAGTCTCTTCAAGAGAGCATTAAACGACATGAGCACTTCCTTGCTTTACAGCTAGGACTAAAACCTGGACAAAAGGTCTTTCTTGTTGATCTACTCAAAGAGTAGAATTTTTAGATCCAAATATTCAATTGACCTCTTGCAAGTTATCTTGAGACTAATGATTCTAGGTTTCGGTTAACACTGTAGGTGTTGGATGTTGGGTGTGGAATTGGTGGACCATTAAGGGAAATTGCTCAGTTCAGGTATTGATAATTGGCGAGTAAAACTATTTTTGCTGGACATTGGTATTGATGTGTTTTGACATCTGTTCTGTCTTTTCACTAGTTGTGTGGATCAATTTGTAAAGCCTCTGCATGTTGTGTCTGATGCTTGATTGGTTTCATTTTAGGATAAAGTTCAATTTCCCTTCATCAAGTTTATGGAATGAGGCACTCCCTTTCCCCTATTTTGGAAACACACACTTACCACCCTGCTATAAGCAAAGCCCAAACATATTCTTTTGACCATTGTCCCTGATGTATTTCAACGTTGATCATACTGCCCTTCTCAAGGTGAAAATCCTAAAAGTTTAAACAAGAGGATTTTATCCCTGAGGAAATGAGTACAACAACCAACTTTCGTCTTCCTCTAGGTTTACAACTATTCATACAACCATGCTCTTGTACCTTAACTGGCATACACTCTCTTTCCTTTCACACTATAAACTGATCCACATTTGCAGTCGTGCGCACATGTGCATTGATATTCATGCATATACATTGATACACGTGCATATGTAATGTATCATGTTTTGTAACACCATTCCTGCAAAATTGATAAGTGAAGGTGTTTGTAGTTGGTGGCATAGATCCAACTTGGAGTTATATCAAAACGAAAATCACAGGCTTGGGATTTGTCATTGCTGGACCATTAGCGCTGGTGGTTATTGGCCAAACAATTGAAGTGAATGGTCTGAAATTAATTGCTCAAAGAGAGATACAGAGAGATTCTTAAAGAGCAGTGGATAATTCTGTGTACTCTTTGTTCGTTATTTAATTCCTCGTGCATTGGAATCAGAGATGGAGCTATTAAGAGGTGGGAGGGACGTGCCACCCCTGGTCCGAGAGATGACACAAATCATGTGTGGGACCCATCTTGAGGTCCCACATAAATGATCCAAGCAGTTCATTTTGCTCGAGATAGTTTTCTAAGTGTGCCTGTGAAAGAGCAGCTCAATCAGATGTCATTAAGGACTTGGTTTATTAATTCAAGTTTTGGTTCCTAAATTTGTAGGGACTAAAAATTGAATAATTTGTGCAAGCCCTTACAAAGATCCGATTGAGCTGATTTCTTATAAGGACCCTTAAAAGAATATTCAACAGAATAAACTGCTTGGATCATTTATGCGAGACCCCCATATGGATTCCATACTGAATTTGTGTTCTACCGGTAAAAGAAGGATAAGAACAAGATGAGTTTAGCGAGAGTTAGATGAGTTTTGAATTATCTCATCTCACAATTCACATGGGTTTAGAGAATTGTTTAAACAGAATGGTCTCCCACTTTGGTCTTTTTCTACACGATTTACGAGATTTGAGAGAACATTGTTGTTGTTCTATATGTTGATAAATAATTAACCTTTGAGCATTTCGATTTTTTTTGGATATTGCAATTGGCAAACCTTATAATACTGTTTACTTTTTTGGGTGTTACATATATTGATCTTTGTGGGTGCCACCTCCGATTCCAAATTCTGGCTCTGTCCCTAATTGGAATATCCCCCTTGCATTGATCCGTTCCGTAATCAATAAGTTGACCCCGTGAACAACCATTAACGAGTCGAAGGGGGGAAGTGCCTTTTTTTCCAAATTGTTTACAGAGTTCCTATCACTTAGGGGATTAGTGGTGTAATTGCTTCAGGATCTATAGTCTTGAATGTATGAATGTTATTCGAATAACCAGTGCAAAAATGTTGGTTGGGGAGCTTGCAGCTGATCTCTCTGCTGAAACCCTAAATGGTAATGATTTCAAACATTTTTATTTTGCTCTACTGGTATTTTCTTGTTGGTGATGACTGAGCTATTGTCCGCTAGTGATCTTTATGTTACGATCCTAGCGTCCCACATGGACAAAGTATGGGCTTAGCCTTATGTATATAAGCTCTTGGGCACCCTCTCCTTGTAAGCCAGTTTTCAAGGGTGAGTTCTACCCATGAGTTTGTATCACTTTATTTGCCTGTAACTTGTGATCACGGCAACATTAGTTCGAATATTATCGCGGTGCTGGTATTAAGACGTGTGTATTGTATGGAAGTTTCAGTTTTATTCAGATGGGCCATGGAAATTTGTACCAGTTAACTAATTTTATTTTCTTAAGAGTTGGTGCTGAGAATCTGTTTGTTGGCTGATTTCTGGCAGCATGACCTCCGTTACTGGATTGAATAACAATGAATACCAAATAACAAGAGGAAAGGTACATGCTTTATGTTTTATGTTTTACACTTTGTTGTTTGGAGATTGTTAGAGTTTGTCCCACATTGAATAATTATAACCTCCAACGCTAGTATATGAGCCTAGGCGGCCTCTCCACTCATTGCCAATTGGTTTTGAGTTGGATGCTTTAACAGTGATGACATGGGAAAAAATCTGAATAGATATTCCCCAATGCAAATAGATTCTTGTTCACCTGTTATTGAATTTTCTGTTATATTTTTTTACTAGGGATTCGTGAACCAAGAATGGTTATAGCACTCTGTATTTGTTTACCTAAATATTTAGTTACTGGGGTTGCCTACTATTCCTGCGAGATAAAAGCTTAGGTGTTTCGGTTATTCTACAGGCACTTAACCGCAAAGTTGGAGTGGACAAGGTTTGCGACTTTGTGAAGGTCCGTAGTTTATAAATAATTCTCATGGTTTTGTTATGAGATTCTTGTTTCCAGTGTTTGATTTTTTTTCCCCCTCTAACTCTTGCAGGCAGATTTCATGGAAATGCCGTTCCCAGACAATAGTTTCGATGCTGTATATGCAATTGAGGCTACTTGCCATGCTCCGGATGCGGTATGTATGTCTGAAACACTTAATATCCTATAAACAGTTTCAGGAGCATTCTTTCAGTATAAAAATATTAGCTTTTGACTTACAGTGAATAAATTTTTTACTTATTTTTGTTTGGCTTTTTTTTAGGTAATTCGGAATGTTTTGTTGGATTGTTCGTTTCCTATTGCCGATTGGTTATACTTCCGTTGCCATCAGAAAGTCCCCTTATTTATTCCCATGTGTTTTCTTTGACATTATCTTCTTGTCTAAAGGTTGGGTGCTACAAAGAGATTTGTAGAGTACTGAAGCCTGGTCAGTGTTTTGCTGCGTATGAGTGGTGCATGACTGATTCCTTTGATCTCCACAACAAAGAACACCAAACAATCAAGGTTTTCAGCAAACACAACCAAGCATACTATAGATAGCATATTTTAGAGTAAGTTGACAAAGATGCTTAATTGTACGTTCAAATTTTTTTGACACAGGCAGAAATCGAGCTTGGTAATGGCCTCCCCGAGGTCAGGGGTACAGGACAATGCCTTGAGGCACTGAAGCAAGCAGGATTTGAAGTGAGTAGCAAATTGCTGGTCTACTAAAAGCTCTCTCATCTGATAGTGAGCAGACCAGACAAAATAGCCAGAATAACGAACTCTATTTTTTCTTTTCCGTCATATGAAGGTAATATGGGAAAAAGATCTTGCAGCTGATTCACCTGTTCCTTGGTACTTGCCTTTGGACAAATATCAACTCTCACTGACTGGCTTCCGTTGTTCGGCTGTTGGACGTTTTATTACAAGAAACATGGTACGTGCTGCCACCAGATTATTTTACTTATATTTTCAGCAGAATAACTGTTAACACGATTTTAGGGGAAGAACAGCAAGTTCAGCTGGAATGCTTCTGCCCAATTCCCCTAAAGTCCACTAGTGAGAGCTTTAAGGCTGTCATGGACAGAACTGGGTTTTATGAACTCTCAATGATAGCACCATGTTTTGAAAAAGTTTTGGTATCGTGTGTGTGTTTTTTTTTGTGTGTATGCACGCGTGTGTAAGGTCATATCTTCTCTATTCCATAATGTTGGCTTGATTAATCGAAGTTTTGATTTTTGTCTCTGAAGGTCGTCGGTTTAGAATATGTTGGACTTGCCCCCAAGGGAAGTCAAAGGGTTCAAGCTTTCTTGGAGAAAGCTGCAGATGGGCTATATGAAGGTGGAAAGTAAGAATTACTCTGACTTGTATTTATTGTCTCTCTCCCCATGCTGGCATACCTTTTATGTAGTAGTCTTCTTTGCTAGTCTTCTTCATTAGTAGTTATGTGTATCTTGTTCTGAGCATGACATCTAAACAACTGGTGCTTAACCCTTCAGGATTTGAAAAGAGCTGTATGGGCTCTTGCTTACCTAGATCCATAAAATCAGTTCTTGGCCTAATCAGCATGATTGGGTGCAGTGTTCTAAAAGAAGGTCTGGAAGGCTGAGTACTTGCCGCCTAGGCGCTAGAAGGCCCCTCGCCGACTAGGCTGACTAGGCGCCGAGTACTCGGCCTTGGAAGCTAGAAGGTGCCTAGCCGGCTTGGGAGCACCTAGCGAGTTTTAGAACCTTGATTGGGTGAAACAGGGTTTGTAGTTTGTTAACTCTGTCCTGTAGTAGTTGATGAAGGAAGAAGTATAGATTATACTACCCTTGCAAGTGTATACATTGCATACATTGCATACTTGTATATACGTACGTAATATACAAAGAGGGTTTTGTGCGCGTGCGGGCATCCATGTGTACCTGTATAGTCGTTTTGGCTCTTTTTGGTTCTTGGGAAAATTAAGGATGGAGGAGTGCTCTTTATACAGGTCGTGTTAGCCATTTTAAGAATTCTACCCTTCAAGTCCTTCTCAAAAGTGTATCTCACTTGTACATATAATTCTAGGTTAAAGTAAACTTCTCAGCTTCCTTGGAATTATCTTTTAAAATGACATGGATTAAGAAAATCATAGCTTCCTTCTTTATGCAATTTAACCTTGTTTTCGATCGTGGAGATCTCTCTAAGTGTTTGACTCATGATCATGTTACTCTGTGTTTCAGGAAAGAAATCTTCACGCCAATGTATTTCTTCCTGGCTAGGAAGCCTCATTTGGATAGCTAATGCCGCATGGATTAGAAATCTCTTGTACTCTGGTGAATCTTCCTGGGAACTCTGCTACTAGATTTCCGTAGCATTTATTCTGTAGTTGCATGTCTGGGTTGTGGTTTCTGAGTTGCTTGTGCGTCCCATTATCCTATTGTATTTTCAAGCTTGCAGTTTAGTTGACTTGTATTCTTTGTTTCGGTCTTTTGTAATCTATGAATGTGGTGTGTGAAATTTTCCTCTATCTTAAGTTAAAAATTTTGTGTCTTCTATTTTGATGTATATCTGGTCTGTAAGTTTTTGGGTTTTCTTTTTTTTTTTTTTCCGTTTTCGGGTTCCGGTTGATCATTCATTGAAAGAACACACAACCAAATTCAAAATCCCCCAAATCACATTTCTTGTTCGACTTTTACAGCGACAAGATCATAAATCTTCCCCCAATCAATCACGTATCATCATTAGACAACAGAATAAAGAATGAACTCGGTGGTGTCAACGCCGCTCCCAACAAAAACCGCCTTAGATCCGCACTTCGCAATCTGGGTGGTAGACGCGTCGACGCCGGCGTTTCTTCCTCCTCCTCCGCGGTGGTCGGTGATGTGGTGGTTTTCGGCAGCGTTTGCCGGTGCCGGTGGGTGGTAGTACATCCTCGCCGTCTGGGGGCAGTGGGAATGGAATCTTGCGGCTATCCTCACCCCTGCGTCTAACAATTCAGCGTATTTCGACATCCCTTTCTTTTCTCTTTCTCTATATGTATCTATATATTGGTATCTGTATGGTTTTGGATTTGTATCTATATATGTAATATTCCTTTCTCTCTCTCTGGGGGATTTTAGAGAGAGAAAGGGGAAGGAAGAGAACTGAGAGAGAGGGAATTATCAGAAGACGTCCCAGAGGTCACAGCCCATGAAACGGCAGAGACGAATTGCGGTGGAGAGAAAGGGAAGGGGCCAATGTTTGCAATTTATAGCTGTCCTCCTCTCTCTCTCTCTCTCTCTCACCGAAACTTGGGAGCCGGTCGGGTATGGTCACGTAGTGTCTAATTGTGCATCTCGACCATCTAAAAGTATTTTGGATTGTCCAGATTTCTTCTCTCTCTTCTTTTTCTTTCTATTCTTTCTTTTTTTGTAATCTAGGCCGTTCAAAATTTTAGACAGCCGAAATACGCGATCAGACATTACGTTTCAAAATTTTAGACAGCCGAGATACGCGATCAGACATTACGTGTCCATACCCAACCAGAACCTTTTCTTGCTTTACTTTCCTTTTCACATTTGTTTATTTTTACAAACCTAATACCCTTTTTGCACATTCTTCTTTTTTGCGGTAATAGTCCCTATAGGTTGGAGTATTTCCATTTCTTTCTTATGTACTCCCTCGGTCTCAATTTATTTGTCTATTTTTGGTGATCGTGTCAATTTTCAATTACTTACATCAAAAAATAATATTGACATAAAAAAATAAGCCGTAAAACTTTTGTTGATAGTCTTCAATTAATTCTATTCTAAAAGATTTTTGAAATCATGAAATTGGGATCGAGAGGGTACTTTTTATGTTTTAACTTGAAAAGAGTAGTTNNNNNNNNNNNNNNNNNNNNNNNNNNNNNNNNTAAGGGGCGTCTTTTGAAAGCAAAACTTATTCATGGTACCAAAACAAAACGCGTCTTATTAAAGTAGCTTGCGTTTAATAATCGCCGCGTGTTGGTGACCGCTTTCGCCGGTCATTTCCATACTTGGATTCATTCTCACTTCAAACTTGGGAGCCCTTCAACTTGCGATTAAGTGCCTATTGTGCCCCCAAGTTCCACACAAAATATCATTATCACCCTCGACTTTTTCCAATCTCCTCTCTGTTTCATACCTATTCTCATTGATTCTTTAGTATTATGCTTCCAGAATGCTCCTTGACATTTCAATATCTGTATTCGTATATTTCAACATTTCCCTTCTTGATATAATAAAAGTAAAGGCTAAATAAAAGTCAGAACATGGGTAATTATTTTGTTCTTAATGGACAGCGGAGTAATTAAGATCATCTCAAGCAGTGGCGGAAGCAGGATTTGTGTCAAGTAGGGGCCGAATCATGTGTATTTTCATATTCATAAAAAAAAATTGTAAGTATTTATTGCTACTTGCGAGGTCCAAATAAGATACGGTATTGTGTGGATGTTTTTAGCCACTGTTAAATTTTATAAAAGCAACCTAAAAAATGATTGATAACTTGAAATTTTAAGAATTTTTACCCCACAAACGAAAAATTTAAGAATTTTTTTCATTCAAAGGACTTGCAGGAGCCAATCCCATAGATTTTTTCGTAATTAACCTAAAAAGCAAGTAGAATACGTGAAAATATTTGGAGCTGTGTATGGACGGGTCCCCCTGCACGGCTGCACCACTTTAGCACCGCCCCTGATCTCAAGGGAAGAAAACCGGGACTGGTCGCATGTCACAACAGGCTTTGGAAGGGACCCATGGTCTAACTGTGCCCCAACATACAGGAAATTGACGTGCCCCAAGTTCATGTCCCATTCCTTGAAAACATACTCCAAATTGTTCTTTAATTTTGTAAATTTTGGTGTGTTCGATAATCTCGTAAAAAATCAAGTCAATCGTGATATATATCAGAAACTCAATAAATGAATCACATTTTTTTTTTCAATACATGAACAACAAATACATTTTAACTCTTACCAAAAAAAAACCCAATACGCTTTAACAAATACATTTTGGATTATTATTTATATTTTAAAATATATAATGTTATATTCCTATATATCAATATGGATGTTTGACATATCTTAATTATTTGTATTAAACATATTTTTTTAAAAATATATATATAATATTGTATTATGGAGCAAGAAATATAAGTATTTTGAAAATGACACTTATGTACGTAAATAGACAAGAAATTTGGGACAGAAATCCCAATGGGTTGGCAAATTAGTCTTGCCTTAGGATTTAGCAGTTTGTTTCCACCTAAGGTTTTATGTTCGATTCTCCTTGCCAGTAAAACTCTTAGGGCCACTGAGAGTTATACCCGGTCATTACCTCCAGGGGCTCCAAATTTAGTCAAGTTGCGCATAAACTGGCCTGAACATTCGATTGTAAGAAATTTTATAAAAAAAAAAGAATGAAATTTGGGACGGAGGGGTATTACACAATGACGAATTTTTGAAGAGAAAGTGTATAGAACATGAAAAGAAAATATAAAAATGAACATGAAAATGTATCCGATAGATGAGATCCGTCCGGTATACTGTTCGGTACACCGGACGAATCTCATCCGTCAAATACCTTTTCATGTACACTAGGGCAATCCCTTTGAATATTAAACAAGTCTTTACTTTTGTTTCAGACCTTCAGTAGTTTTTCAGAGAACCCATTGTGGTCCCCTTTAGCGGCCCATCCACCGAAAGCCTGAATTAAGTTCTACCTCCACTAGTTGACCTAATATCCACAAATTAAGGAAGGAAAAATTATTCAAGAGAAAGGAAAGGAAAGGAAAGAGGGTTTAAAGAAAGTCAATAAAAGATCTTGAGAATGAACTTGAAGAATTAATTGGAATTAAATCCTTTGTTCTGAAAATTCTTCACCTTGTTCGGTTTGGATCTCAAGTCCACCCTGCACGGTCCCCAATAAAATCTTCTTTCTATCTCTCTCTACTCATTAATCCCAAAACCTCTCTCAAAACCGAACAAAGCCAAGCTTTATTCCTCTGTTTCAGTGCACTTCCGGTCATTGGATCACATGAAAATTTTGAAACAAAATACAAAAAACATACCATTCAACAACACAAAACAATTCGGCACATAAGCGCACCATATTTTGGGCAAAGGGGGATCTCAGGCGGACTTGATTTGGGTTTTTAGAAAAGGAGGGCTGGGGCTGGCCGGCTGGGACCACAACAAGATGTTGTGCCCTTTCTATGGTCTCTATTTTGAGCCTACAAGACTTGTACTCCCCCCGTTCTTAATTGATAGTCCTTTAAGATTGTGTCATTCTTCAAATAAAAAAATAAAGTACTTTCATGTAAAATTTTCTGAATTTTTCCGCACCGAATTAAAGTACTAGATTAGTACTTTCATTCGGTGCGAAAAAATTCGATCTAGTACTTTAATTAGGTGCGAAAAAATTCGAAAAAAGTTATACACGGAAAAAAAGCATTCAGGTAAATAAGGGTAATGGATTCCTGTGTAGTGTTTCGCTCTAAATTAGTAACAATAATCGCTCTTTACCTGTATCGATGGGAAAAAAGAAGTGGAAAATAACTTTTTAATTTTAGGTCCAACCCATTTTGAACCCATGTAAACCCACGTGTATATGGGTTAATATGGATTCACCCATTTATAACTCATATTTTAATATAGGTGGGTTGGGTTGGGTTTTGAGTGGGTGAATTTGGATGGGTTCATGGGGTTGGGTTAGAAATTGTCACCTCTACTTATGTATTTTAATTTATTATTTAATGGAGGTTAATTGAATTTCTTTGAAATACTCGTAAACAGGTAATAAGTACATGTGTGCGTTAGTTATTCAATGAAATAAATAAATAAATTCGCATATTATATTGCATTGGACTCAAAATAATCGAGTTGTATAGAGAAGGATACGAGAATACAGCGATGTACAAATAGTATATTCTCGAATGAAATCTATATATCTCTTTGTTCGGCCTCCCCGTGTCAAAGTTCTAGCTCCGCCACTGATTAAATCTCCAATATATATTATGTGATGCTCATAACACCATTAGTCAATTGAAGGGAGAGATCTCAATACACCTCATAATATCACTAACAAGCAAAGTTCAAAATATATTACGTAAGTTCTTTTTTTTCTTTTTTGGCATCAACTTATTCAACATTTTACTGCTAATAGTTTATGTGTAGGTCAAAGTTCCATAAATATGGTGAAATATTTACCATTCTCCTTTCCACTTTGTTAGGATTATCTTCTTAAAACAAAGTTGTCAAAATTGACCTCTACAGAAATGCATGGACCGTGGTTGAGTAATTGACTAATTAGTAAATTATATCCTTGAATTGGTTGGTCCTCTTTTCTCCATATATTAAAGCATAATCGCATGAGCAGTTGTGAAAATTCCACGATTCACGATGCATAATATTTTGCGATTCGATACTTTTAGAGGTAAAAGTACTGTTGGATTTCGGCTCGATATTATTACTGATTGTGTGACGAGCACGCAAGCGTCTAGTGCCGGCTCATTAGTTTTAGAAGCCTGCAACGGACTCATTAGTATATTTATCATTGTACAAAGTTACATATATAGTCATCTAAAAAATAAACAACCATTACAATATATAAGAGGTTTAAATGTTTGGGGGTCCATATTTTTTGTGTTTTGGGGGGCCCCAAAGCGGTGACTTCATGAGACGGCCCTGATCTGATGAGCGCCCGACTTTGTTTATTGCTAATCTTTTGGTCAATTTGAACTTGGTTCGGACTTTCTAGAGCGTTGTTTTGGATGTTGGTGTAGAACTTTTAGTGCAAGAATATTAGTTTTGGGCAAGAATTAACTTTCAAAAAAGAATCTTTAATTAAGGATTTCATGACTCTCTCTGAATTAAGGTAGGACTTCTAAGTTTCTTGTAGACTCCTACAGGATCCTTAGTAGAATGGAACCTTAATGAATTAAGGTTGAAACTAGGTTTTGTGGGACTTAATGAGAAAATGTTTTAGTTGGGAGGTTCCAATGGGGTTATGGTGAGTTTGTCTCTTGGTGAGAGTTTTCTTTATTACCATTGCAGCTCTTCAAAGTTTTGTTCTATTCACAAAGAAGTATTTGGTGCACCCTTTACAAATTTTTGTAGCTCATTTTATGGCTTTTTGTGGTACTTTTACGAAGTTTCGTGGACCGTTATATGAATTTTATAAGTCGAATCTATGAATATTGTAGTCAAAGAAGGGGGCACAAAGTTATGTAGGTACACAACTACCAACCACACCTCCAATCACACACATCTCACATGCATATGGGTCCCACCTCATATTTGAGGTTTAAGATTCATATTCATGTGAGAGGCGTGGTTGGAGGTGTGGTCGGTAGGGGTGTATGTAGCACTGTTGATAATATCAATCAAAACAGGTCTAAAACACACAGCTGCATTATAAGGACACCTAAGACCTTGCACCCAGGGGCAAATCCAGAGTAAGCACAGAAAAATTACCCAAAATAGGGTCCACAAGTAGAAGGAAGTATTATTATATTAAATAGCCTAAAATCTACTTGGATTTGATGTATTCCAGTGGTAAAATATTTGACATCCAGGAATATGATACAGGTTTGGGTCTGGGTGCTCTCCCCCTTATTTTTAGTGTTTAAATGGTTTTAAAAAAAAACTCTCCATGAAATTTTTAGTGTGTTGAGACTCGAACTCACACTAAAACTAACTTGTGTAGCACTGGCCCCACAAAGCGAACTATACGATATGGAATTAGTACTCTAAAACTAGTCTAGCCTTCAATGGGTACTGAAAAATTATAAGAAGGTGAAAATAAATAAATGCACCCCAGAAGTCCATACATTCAATTGCTCCAGATGAGATGATCCTAACTTCATAACCAGATCTGCATTGCTCCAAACTCAGCATATCATTCAAACAGTCGTGTCATGTTCCGCGTAACATAAGCAAAAAGAAAAGACAGAATGAGCTTCTAAAATGTATTTCGCCAACCAAGAAACAAAACATGGCAGGTCCTCCGATGACAAAAAGTTCACGTGACCTTCGACGACAAAAGTTCACATGAACAAGAAATATCCACTGAGTTTCGTTTCGTCCCGTCCTAGAAACAATGCCCAAGAGATAATGACAAAAGTTCACCTGAACAGGAGGTGAATTGGGCCAGAAAAGGAGTCAATATTGGTGAGGATCCCATGCCGCTCGGGTCCGGCAAGGTGAACCCAACTGTTTCTGAGCTTGGTAATCAAAGGCAGGATACTACCATAACTTCATATCCTGTGTTTCATGTAATCAGCATTTGCGATTTCTTCATCTTGCACAAGTAGAACTGCCGTTCTCTTTACACGTCCTTCAACTAAAAGATTCCAAAGTGAGAGACAAAAGGATTAACAATCAAATCTTGCCTTCAGAATTCAAAACAAAATATACAAAAACTCATGCAGTGATTACTACAAGTGCATAAGAACGACCTTCAAAATAAAACAAGAATACAGCAGAACACATGATTCAGAAATAGACCTAGCTTAACAGACCCTAACCATCCAATAATCAGAGGAAACCATTAGACTCACTCAATAATCAGAGGAAACTTAAAAAGAACTCTGTGCATTTTTACCCATTCATATTGATCATGACTTGCACACAGTAGCAATTTCGGATCGTAATCCTTCTCATTTCCACACACAGAAAAAAGATCGTTTTCTTAAGCTATGATTGAAGGCAATAAACAGAAGAAAGGGAAGCAAACTAGCCATACATATATACTTGCTCAAACCTCTTTGAAAGCAATAAATGCAACAACACACCCACCCATTAACTGCACACATATGCACCAGATTATTTAGCTATGACTAGGATGGAGCACTGACACGAAATTAAAAATATTAAGCAAGGGATGTAATATTAAGAGATGCCAACAAATTGGGTGAAACACTTTGCTCCGAGAGAAAGGATTACAGAAGTACTTCTCGTATTACAATAACCCGAAAAGGGTGGCGGTAACGCAAATGCTCTGTGCCATGTTCATACATTCAGAAAATTGTTGATTCACAGTTTGCAAAAGAACAAACAAACGATTATTTCCTCACCATGAACAGTTTAATCATAACTTCTTAAGGAAAAATGTAACGGTTTGGGTCGGGCATAATGCTACATCTCTTGTAAGCATTTCTCGAACAGTTATAATACACATTGTCTCCATTAGAACTGAACAAATCATGGGTCAGTATTAGATTTCTGGAAATCAAAAATTCCCAAAGGAATGAGATACGGAGCGGAGGGAGAGAGAGAGAGAGCACCCAACGGATGAGTGGAGTGATGCTCGCAGAAAACGTTTAAAATGCCTTATTAATCTGGAAATCATTTTCCGGCTTATTTGGTTCATTTTTGGTTTGACTAGGAGAAAGTTTTTTTGTTGACTTCAGTTTTCCAATTATAACAATCACAGGAAAATGTTTTCCATGGAGAATCTTTTCCCATAAAATCGCCCAAAATAAACGGACCATTAACCCAAATATTGCAAACCATTTTGCACACGACTGCTACGACCACCTAAACAGCAGGACTCTCCCTCCCTCCCTCCCTCTCTCAAGCTTCCACTCTTCTTCTTTGGTATCTCATGAAGCTATCTAAACTTGCTCTCAAATTCCCGCAACCATCGTCAGACGACCGCCAACGTAAATCTTGTAAATTCCTATTCTTCCACGATCTGATCTCACAGTTGCTGAATTTTGGATTTCCCACAATTCAATGGAATGGTTTTTGGTGAATAAAAAGTATGAATTCTACCTATTCGAAGAAACGAAAGAGACGCATTCACAAATTGTTTTGTGCAATCTAGAGCGTGGGATAAAAACTGCCTCTCATCCTTCTCTTCTCTCATTATGATCAGTTTCATAGTCGAATATCTGGTGTGGAAAATTGAAGGCACAAATACCTTCTTCTATCAGATTGTTTGCCCCAAACGGAGTAGGCACCTTTCTTTCCTCTTTGTCACTCTCTCAAAAAAAAAAAAAGAAAGAGAGAGAGAGATTTCTGAGTGCTTACCATTTGGTGCATTCGATGTTCCATTCCACTTATTTCTTGAGCATCCCTTTGCGACTGAACTAATTCAGGTACCAAATGCATGTCAATTACTTCACCTACGTA -3’
