# Supplementary material for: An invaluable transgenic blueberry for studying chilling-induced flowering in woody plants
Source: BMC Plant Biol. 2018 Nov 1;18:265. doi: 10.1186/s12870-018-1494-z (PMC6211425; doi:10.1186/s12870-018-1494-z)
Supplement: Supplementary file 7 — Table S5. Primers used in this study. (DOCX 18 kb) [file 12870_2018_1494_MOESM7_ESM.docx]

**Table S5** Primers used in this study.

Primers for qRT PCR

| Oligonucleotide name | Sequence (5’ to 3’) | Comments |
| --- | --- | --- |
| VcTFL1_Fwd | GTTGGAAGGGTGATAGGAGATG | c22179_g1_i1 |
| VcTFL1_Rev | GCCTGGAATGTCGGTGATTA | c22179_g1_i1 |
| VcARP6_Fwd | TGGTAGGTATGGGTCAGAAAGA | C49456_g2_i2 |
| VcARP6_Rev | GCTGTGGTCACAAAGGAGTAG | C49456_g2_i2 |
| VcFD_Fwd | CGGAGTCGGAAGTCAGAAATAC | c75407_g1_i2 |
| VcFD_Rev | GAGTGTCTCTTGGGAAGTTGAG | c75407_g1_i2 |
| E1F | CAGTGATGGCGAGACTCGTA | c96767_g2_i12 |
| E1R | AGCACCCATTTGACAAGACC | c96767_g2_i12 |
| C94438_g3_i2: Eukaryotic translation initiation factor 3 subunit H FWD | GAGAGATTCAGATGCCCAGAAG | VcEIF |
| C94438_g3_i2: Eukaryotic translation initiation factor 3 subunit H REV | GGACAATGGATGGACCAGATT | VcEIF |

Primers described by O'Malley et al (2007).

| Long strand of adapter 1 (**LSA1**) | GTAATACGACTCACTATAGGGCACGCGTGGTCGACGGCCCGGGCTGC | Long strand adapter 1 is used in combination with short strand Eco and Hind adapters |
| --- | --- | --- |
| Long strand of adapter 2 (**LSA2**) | GTAATACGACTCACTATAGGGCACGCGTGGTCGACGGCCCGGGCTGTGC | Long strand adapter 2 is used in combination with the short strand of adapter Ase |
| Short strand of adapter Hind (**SH**) | 5’-Phosphate-AGCTGCAGCCCG-amino C7-3’ | 5’ phosphorylated and 3’ C7 amino modification.  HPLC purified |
| Short strand of adapter Eco (**SE**) | 5’-Phosphate-AATTGCAGCCCG-amino C7-3’ | 5’ phosphorylated and 3’ C7 amino modification.  HPLC purified |
| Short strand of adapter Ase (**SA**) | 5’-Phosphate-TAGCACAGCCCG-amino C7-3’ | 5’ phosphorylated and 3’ C7 amino modification.  HPLC purified |
| Adapter primer 1 (**AP1**) | GTAATACGACTCACTATAGGGC | Primer for first PCR |
| Adapter primer 2 (**AP2**) | TGGTCGACGGCCCGGGCTGC | Primer for second nested PCR for Eco and Hind adapters |

Primers for detection of T-DNA borders.

| Oligonucleotide name | Sequence (5’ to 3’) | Comments or Tm |
| --- | --- | --- |
| RB3 | GCAAACTAGGATAAATTATCGC | 58.8 |
| RB4 | GGATAAATTATCGCGCGCGGTG | 72.6 |
| RB5 | GCGGTGTCATCTATGTTACTAG | 58.4 |
| RB6 | CTAGATCGGGAATTAAACTATC | 55.8 |
| RB7 | GTGTTTGACAGGATATATTGGC | 60.2 |
| RB8 | CGGGTAAACCTAAGAGAAA | 56.5 |
| LB3 | CAGTACTAAAATCCAGATCCC | 57.3 |
| LB4 | ATCCAGATCCCCCGAATTAA | 64.5 |
| LB5 | CCCCCGAATTAATTCGGCGTTA | 71.0 |
| LB6 | GTACATTAAAAACGTCCGCA | 60.5 |
| LB7 | CGCAATGTGTTATTAAGTTGTC | 59.2 |
| LB8 | CTAAGCGTCAATTTGTTTACACC | 61.7 |

Primers for confirmation of the DNA sequence at the insertion position

| Oligonucleotide name | Sequence (5’ to 3’) | Comments or Tm |
| --- | --- | --- |
| C1F | GGTATGGAGGATTTCCCAAG | 58.31 |
| C1R | TTGACAAAATGGGGTAAAGC | 57.61 |
| C2F | CCCATTTTGTCAATTGTGGT | 58.17 |
| C2R | GATGTCAGGTGCTGCTTCTC | 58.54 |
| C3F | CTTGGGGATAAGAGCAATGA | 57.76 |
| C3R | TAGGGCATGACCCAAACTAA | 58.09 |
| C4F | GAGCTGCAGAGATGGGTCTA | 58.13 |
| C4R | AACGAAGGAAGGAGGAGAGA | 58.05 |

Primers for extension of the DNA sequence at the insertion position

| Oligonucleotide name | Sequence (5’ to 3’) | Comments or Tm |
| --- | --- | --- |
| D1F | GGTATGGAGGATTTCCCAAG | 58.31 |
| D1R | ATCCGCGCAATATTTTACCA | 60.30 |
| D2F | TGGGGATAAGAGCAATGAGG | 60.03 |
| D2R | ATCCGCGCAATATTTTACCA | 60.30 |
| E1F | CAGTGATGGCGAGACTCGTA | 60.01 |
| E1R | AGCACCCATTTGACAAGACC | 59.97 |
| E2F | CGTAGCTGCTAGCACAGTGA | 57.99 |
| E2R | AATGCTCTTCGGCTGAATTA | 57.61 |
| F1F | CCTTCCTTCGTTTCCTCTTC | 57.98 |
| F1R | GCAGCTACGGGTGTGTACTT | 57.91 |
